# *In Vivo* Colonization with Candidate Oral Probiotics Attenuates Streptococcus mutans Colonization and Virulence

**DOI:** 10.1128/AEM.02490-20

**Published:** 2021-01-29

**Authors:** David J. Culp, William Hull, Matthew J. Bremgartner, Todd A. Atherly, Kacey N. Christian, Mary Killeen, Madeline R. Dupuis, Alexander C. Schultz, Brinta Chakraborty, Kyulim Lee, Deneen S. Wang, Verisha Afzal, Timmy Chen, Robert A. Burne

**Affiliations:** a UF College of Dentistry, Department of Oral Biology, Gainesville, Florida, USA; University of Manchester

**Keywords:** *Streptococcus*, oral microbiology, diet, qPCR, biofilms, antagonism

## Abstract

A collection of 113 *Streptococcus* strains from supragingival dental plaque of caries-free individuals were recently tested *in vitro* for direct antagonism of the dental caries pathogen Streptococcus mutans and for their capacity for arginine catabolism via the arginine deiminase system (ADS). To advance their evaluation as potential probiotics, 12 strains of commensal oral streptococci with various antagonistic and ADS potentials were assessed in a mouse model for oral (i.e., oral mucosal pellicles and saliva) and dental colonization under four diets (healthy or high-sucrose, with or without prebiotic arginine). Colonization by autochthonous bacteria was also monitored. One strain failed to colonize, whereas oral colonization by the other 11 strains varied by 3 log units. Dental colonization was high for five strains regardless of diet, six strains increased colonization with at least one high-sucrose diet, and added dietary arginine decreased dental colonization of two strains. *Streptococcus* sp. strain A12 (high *in vitro* ADS activity and antagonism) and two engineered mutants lacking the ADS (Δ*arcADS*) or pyruvate oxidase-mediated H_2_O_2_ production (Δ*spxB*) were tested for competition against S. mutans UA159. The A12 wild-type and Δ*arcADS* strains colonized only transiently, whereas the Δ*spxB* strain persisted, but without altering oral or dental colonization by S. mutans. In tests of four additional candidates, Streptococcus sanguinis BCC23 markedly attenuated S. mutans oral and dental colonization, enhanced colonization of autochthonous bacteria, and decreased the severity of smooth surface caries under highly cariogenic conditions. Results demonstrate the utility of the mouse model to evaluate potential probiotics, revealing little correlation between *in vitro* antagonism and competitiveness against S. mutans *in vivo*.

**IMPORTANCE** Our results demonstrate that *in vivo* testing of potential oral probiotics can be accomplished and can yield information to facilitate the ultimate design and optimization of novel anticaries probiotics. We show that human oral commensals associated with dental health are an important source of potential probiotics that may be used to colonize patients under dietary conditions of highly various cariogenicity. Assessment of competitiveness against the dental caries pathogen Streptococcus mutans and impact on caries identified strains or genetic elements for further study. Results also uncovered strains that enhanced oral and dental colonization by autochthonous bacteria when challenged with S. mutans, suggesting cooperative interactions for future elucidation. Distinguishing a rare strain that effectively competes with S. mutans under conditions that promote caries further validates our systematic approach to more critically evaluating probiotics for use in humans.

## Abstract (precis)

Our results demonstrate that *in vivo* testing of potential oral probiotics can be accomplished and can yield information to facilitate the ultimate design and optimization of novel anticaries probiotics. We show that human oral commensals associated with dental health are an important source of potential probiotics that may be used to colonize patients under dietary conditions of highly various cariogenicity.

## INTRODUCTION

Dental caries remains a highly prevalent disease and global health problem. Caries results from repetitive and/or prolonged demineralization of tooth enamel driven by exposure to low pH from organic acids produced by acidogenic oral bacteria during fermentation of dietary carbohydrates (1). Counterbalancing acid production and demineralization is alkalinization of oral biofilms by certain commensal bacteria, which in addition to salivary buffering and delivery of supersaturated calcium and phosphate ions, promotes enamel remineralization (1). Furthermore, dental caries is a complex polymicrobial biofilm disease associated with competitive interplay between exopolysaccharide matrix-forming and acidogenic opportunistic pathogens with health-associated oral commensal species (1). With increasing exposure to dietary carbohydrates, such as sucrose (i.e., table sugar) or the high-fructose corn syrups used in many soft drinks and processed foods, the progressive production by cariogenic pathogens of organic acids and of a surrounding insoluble extracellular matrix composed of specific glucose polysaccharides (i.e., glucans) results in a microbial community shift that favors colonization of tooth biofilms by acidogenic pathogens at the expense of nonpathogenic and less acid-tolerant (aciduric) commensals (1). However, a number of commensals in dental plaque are associated with the health of dental surfaces, including several *Streptococcus* species (e.g., S. gordonii and S. sanguinis) (2–4). *In vitro*, commensals can combat cariogenic pathogens, such as the acidogenic and aciduric human caries pathogen S. mutans, by hindering its growth and viability through one or more mechanisms, including production of H_2_O_2_, the secretion of bacteriocins and other antimicrobial compounds, and interference with intracellular signaling pathways (1, 5–8). In addition, many oral commensals produce ammonia, either through the action of urease enzymes on urea, which is present at millimolar levels in saliva, or by catabolism of arginine to produce ornithine, ATP, and CO_2_ plus two molecules of ammonia by the three-enzyme arginine deiminase system (ADS) (9, 10). ATP generation is thus beneficial bioenergetically to ADS-containing commensals. Micromolar levels of arginine are present in ductal saliva, and arginine is abundant in salivary peptides and proteins (11). Generated ammonia counteracts acids from acidogenic bacteria to promote dental plaque pH homeostasis and tooth remineralization, while assisting less-aciduric commensals to survive, grow, and compete against S. mutans (9, 10, 12). Collective evidence strongly supports a positive correlation between ammonia generation by commensals within dental biofilms and a lower incidence and severity of caries (13, 14). Also, delivery of 1.5% arginine as a prebiotic within dentifrices was shown in clinical studies to reduce caries onset (15) and decrease the increments of decayed, missing, and filled (DMF) teeth and DMF surfaces (16, 17).

A strategy gaining acceptance to support the health of dental tissues is the use of probiotics, administering to the oral cavity bacterial strains that are beneficial to the growth and maintenance of healthy biofilms but that further suppress colonization and virulence mechanisms of cariogenic pathogens (18). Current evidence of probiotics preventing dental caries primarily incorporates conventional strains used in treating gastrointestinal disorders (e.g., *Lactobacillus* spp. and *Bifidobacterium* spp.) but with inconsistent results in pilot clinical studies based mostly on salivary levels of *mutans* streptococci, although a handful evaluated caries incidence (19, 20). Furthermore, oral colonization by probiotics is frequently transient and limited (19, 21). Although there is abundant *in vitro* physiological and molecular data on competition between a cariogenic pathogen and an oral commensal (1, 5–8), including potential probiotic strains (5, 18, 22–24), there are very few reports testing oral commensals as probiotics (24–27), even though these species are naturally adapted to colonization of specific oral sites, including dental biofilms (28).

A putative probiotic strain must contend *in vivo* with bacteriostatic, bactericidal, and clearance mechanisms of saliva (29) and further cope with the intermittent availability of host dietary components for its own metabolism. A probiotic also must contend with symbiotic and competitive interactions with the widely diverse nonpathogenic and pathogenic microbial inhabitants of the soft tissues of the oral cavity and dental biofilms (5). *In vivo* models are therefore key to further advance caries research by providing a framework to assess a putative probiotic in its interactions with the host, in addition to commensal and pathogenic oral species. Mice have been used in caries studies (30–37) and are susceptible to colonization by human commensal streptococci (38–42). Knockout mice additionally allow interrogation of the impact of host factors, such as specific salivary constituents, on colonization by commensals and the induction of caries by oral pathogens (43–45).

To identify and test putative probiotic strains in the prevention and treatment of caries, we have taken a systematic approach, targeting commensal streptococci, which represent an abundant genus found in healthy dental plaque (28). First, we recently isolated 113 *Streptococcus* strains representing 10 species from supragingival dental plaque of individuals free of clinical lifelong dental caries. Each strain was tested *in vitro* for two specific phenotypes, production of ammonia from arginine catabolism by the ADS and direct antagonism of colony growth by pathogenic S. mutans UA159 (18), a highly virulent strain that displays great stress tolerance compared to other clinical isolates of *mutans* streptococci (46). In this study, 12 potential probiotic strains representing 7 species of streptococci with various capacities to express the ADS and antagonize S. mutans were further evaluated for oral and dental colonization *in vivo* using a previously established mouse caries model, but with extensive modifications (43–45). Modifications included incorporation of strain-specific quantitative PCR (qPCR) assays to examine colonization by each strain of inoculated human oral streptococcus and a newly developed qPCR assay to evaluate colonization by the population of murine autochthonous bacteria. Novel healthy diets of various cariogenicity, with and without addition of the prebiotic arginine, were incorporated to identify strains that colonize well under each dietary condition. The competitiveness against S. mutans of select human commensal strains and otherwise-isogenic deletion mutants was then examined *in vivo*, and the impact on smooth surface and sulcal caries was assessed. Collective results highlight the importance of systematically evaluating candidate probiotic strains *in vivo* to more critically evaluate attractive probiotic candidates and functional genetic elements for further study. More specifically, we identify a strain that under highly cariogenic conditions promotes colonization by dental autochthonous bacteria, attenuates colonization by S. mutans, and decreases severity of smooth surface caries.

## RESULTS

### Colonization by 12 human oral commensals.

Colonization by 12 strains of human commensal streptococci with various levels of ADS and S. mutans antagonism, as assessed by zone of inhibition (18) (see Fig. S1 in the supplemental material), were initially tested *in vivo* using a mouse model, as described in Materials and Methods. Our initial goal was to compare among these 12 strains their recoveries from dental biofilms and from recurrent oral swabs. As demonstrated later in Results, oral swabs provide a measure of colonization within saliva and the oral mucosal pellicle (47), thus representing potential reservoirs for subsequent colonization of dental biofilms. Recoveries of bacteria released from swabs or after sonication of molar teeth to disperse bacteria from dental biofilms were determined by strain-specific qPCR assays of genomic DNA, thus avoiding genomic integration of an antibiotic resistance cassette, which could unpredictably alter the behavior of a strain *in vivo*. Also, estimates of recovered total bacteria were determined using a novel qPCR assay targeting conserved regions of the ubiquitous single copy gene, *rpsL* (30S ribosomal protein S12) (48), rather than by CFU on blood agar plates (44, 45); the latter may overlook bacteria rendered nonviable during molar sonication. Subtraction of recovered genomes of inoculated strains from total recovered genomes thus estimates the population of recovered murine autochthonous bacteria. Colonization was compared among two primary diets; a diet representative of the average healthy American diet with 11.5% added sugar as sucrose (49) (average diet) and a highly cariogenic diet containing 37.5% sucrose, plus providing mice with 4% (wt/vol) sucrose in their drinking water *ad libitum* (high-sucrose diet). We reasoned that a probiotic must effectively colonize the oral soft and hard tissues irrespective of the cariogenicity of an individual’s diet, as diets will vary among humans and from day-to-day for a given individual. Two other diets were created by addition of 1.5% arginine to each primary diet to determine whether arginine provided as a prebiotic influences colonization by a candidate probiotic strain. An increase in colonization with added dietary arginine would suggest that some minimum level of ADS activity may be required for a strain to more effectively counteract acids produced by members of the autochthonous bacterial population and may therefore require arginine as a prebiotic to be competitive against S. mutans. Conversely, decreased colonization with added arginine indicates that simultaneous use of arginine as a prebiotic may be contraindicated. Diets were based on the nutritionally balanced diet, AINS-93G (50), rather than the commonly used cariogenic diet, Diet 2000, which is nutritionally deficient in vitamins and minerals that likely influence its cariogenic properties (51). The constituents of each diet are given in Table 1.

**TABLE 1:** Experimental diet ingredients (% total dry weight)^*a*^

Ingredients	High-sucrose diet	Avg diet	High-sucrose diet + Arg	Avg diet + Arg
Sucrose	37.5	11.5	37.5	11.5
Corn starch	24.0	24.0	24.0	24.0
Casein	20.0	20.0	20.0	20.0
Maltodextrin	3.2	29.2	1.7	27.7
Arginine			1.50	1.50
Total protein	17.7	17.7	19.2	19.2
Total carbohydrates	62.1	60.8	60.7	59.4
Total fat	7.2	7.2	7.2	7.2
				
Envigo catalog no.	TD.160810	TD.160809	TD.160812	TD.160811
Water additive	4% sucrose		4% sucrose	

a Diets are modifications of Envigo’s AIN-93G purified diet in which corn starch is decreased 40% and replaced with maltodextrin or sucrose. All diets contain the following ingredients in addition to those listed above (% total dry weight.): 7% soybean oil, 5% cellulose, 3.5% complex mixture of minerals without sodium fluoride, 1.5% complex vitamin mixture (AIN-93-VX; catalog no. 94047), 0.3% added l-cystine to balance amino acid contents, 0.25% choline bitartrate, 14 µg/g tert-butylhydroquinone as antioxidant. The vitamin mixture accounts for 1.5% of the sucrose in each diet. In the diets high in sucrose, mice were also provided with 4% sterile sucrose water *ad libitum*.

**FIG 1:**
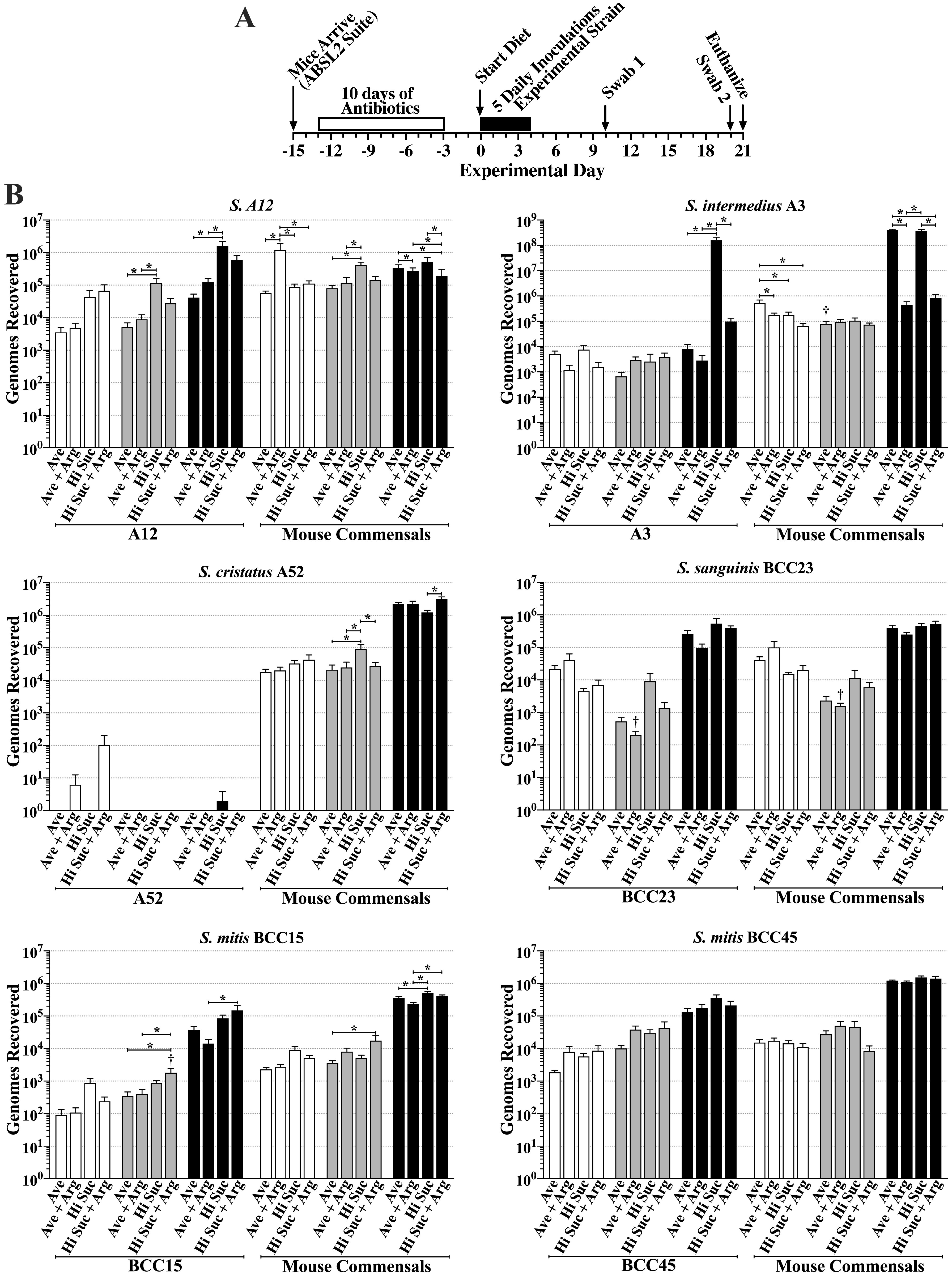
Colonization of the oral cavity and mandibular molars of mice by isolated human oral commensal strains when fed one of four different diets. Diets include the average diet (Ave), the average diet + 1.5% arginine (Ave + Arg), high-sucrose diet + 4% sucrose water (Hi Suc) and the high-sucrose diet + 1.5% arginine with 4% sucrose water (Hi Suc + Arg). (A) Timeline of key events in the experiment. (B) Results for each indicated inoculated strain and mouse oral commensal expressed as genomes recovered as determined by qPCR (mean ± standard deviation [SE], *n* = 10 mice per group). Results are from six experiments, each testing a pair of human commensal strains. Each experimental pair of strains is presented side by side. Oral swabs 1 (open bars), oral swabs 2 (gray bars), and sonicates of mandibular molars (black bars) were each taken at the times indicated in panel A. *, *P* ≤ 0.05 by one-way ANOVA with Tukey’s multiple-comparison test. †, *P* ≤ 0.05 versus the same diet in swab 1, by one-way ANOVA with Tukey’s multiple-comparison test.

**Oral colonization.** Of the 12 strains 2 each were examined in one of 6 separate experiments. Figure 1A shows a timeline for each experiment. As shown in Fig. 1B, a striking outcome of the colonization results is the nearly complete inability of S. cristatus A52 to colonize the oral cavity and molar biofilms, whereas all other strains colonized, albeit to various extents. Oral colonization, as assessed from oral swabs, ranged from 10^2^ genomes for S. mitis BCC15 to 10^5^ genomes for BCC32 and A12. There was only a single example in which oral colonization was significantly increased by added arginine (i.e., S. gordonii BCC32), whereas S. mitis BCC08 displayed decreased colonization, though in both cases, these differences were observed only with the high-sucrose diets and were inconsistent among swabs at experimental days 10 and 20. Interestingly, S. gordonii BCC32 has very high ADS activity compared to nearly undetectable ADS activity in S. mitis BCC08 (Fig. 1S). However, oral colonization by all other strains with either similar or higher ADS activity were not impacted by arginine. Collective results suggest oral colonization is mostly independent of added dietary arginine and a strain’s ADS activity.

The only example where increased sucrose showed a trend toward impacting oral colonization was for A12, where recoveries from oral swabs were consistently at least 5-fold higher in the high-sucrose diet than the average diet at day 10 and day 20. In only a few cases did oral colonization under the same diet change significantly from experimental day 10 to day 20, suggesting oral colonization mostly achieved steady-state levels by day 10.

**Dental colonization.** Five of the twelve strains exhibited relatively high levels of dental colonization with all four diets, ranging from about 10^5^ to slightly above 10^6^ genomes (i.e., S. sanguinis BCA8, S. sanguinis BCC23, S. mitis BCC45, S. mitis BCC08, S. mitis BCA12). Only *S. cristatus* BCA6 exhibited a significant increase in dental colonization with both high-sucrose diets, whereas six strains displayed increased colonization with at least one of the high-sucrose diets (i.e., S. intermedius A3 and A12, *S. cristatus* BCA6, S. gordonii BCC32, S. mitis BCC15, and S. oralis subsp. *dentisani* BCA1). Interestingly, S. intermedius A3 stood out by exhibiting 4-log higher molar colonization with the high-sucrose diet versus the average diet. S. intermedius A3 and S. oralis subsp. *dentisani* BCA1 displayed significantly lower dental colonization with addition of arginine to the high-sucrose diet and a trend toward lower colonization when arginine was added to the average diet. None of the other strains exhibited a significant increase in molar colonization when arginine was added to either diet.

**Murine autochthonous bacteria.** Murine autochthonous bacteria recovered from oral swabs were in nearly all cases consistent among the four diets and between experimental day 10 and day 20, suggesting resident bacteria are at steady-state levels by experimental day 10, regardless of diet. With respect to colonization of molars, mouse commensals also displayed few and relatively minor differences between diets, especially when comparing a high-sucrose diet to its respective average diet. A notable exception was mice inoculated with S. intermedius A3, in which recoveries were extremely high from mice fed the average and high-sucrose diets but were dramatically reduced by addition of arginine to each diet, mirroring in large part colonization by S. intermedius A3. Furthermore, mouse commensals recovered from dental biofilms of mice inoculated with *S. cristatus* A52, which failed to colonize, were consistent across diets (10^6^ to 2 × 10^6^ genomes) and not significantly higher than those seen with mice challenged with the other 11 colonizing strains of human commensals (10^5^ to 10^6^ genomes). These results suggest the total population of resident dental bacteria were only moderately impacted when mice were infected with a human commensal, regardless of diet. In all cases, mice fed each of the four diets gained equivalent body weights during the experiments (Fig. S2A), an indication that the overall health of the mice was not compromised by a colonizing human strain or diet.

### Examination of *Streptococcus* sp. strain A12 and mutant derivatives thereof.

The novel strain *Streptococcus* sp. A12, a relative of Streptococcus australis based on comprehensive phylogenomic analyses (9), has been studied *in vitro* due to its high levels of antagonism against S. mutans and ADS activity (Fig. S1) (5, 7, 9, 52). One mechanism used by A12 to inhibit growth of S. mutans *in vitro* is H_2_O_2_ production through pyruvate oxidase, encoded by *spxB* (9). Also, production of ammonia from arginine via the ADS is predicted to help counteract oral biofilm acidification by S. mutans to promote pH homeostasis *in vivo*, thus creating an environment less favorable for the emergence of aciduric organisms. We therefore set out to determine whether A12 interferes with colonization by S. mutans or colonization of autochthonous bacteria *in vivo* and if either outcome was affected by elimination of the ADS or pyruvate oxidase, using the Δ*arcADS* mutant, lacking ADS activity or Δ*spxB*, respectively.

**FIG 2:**
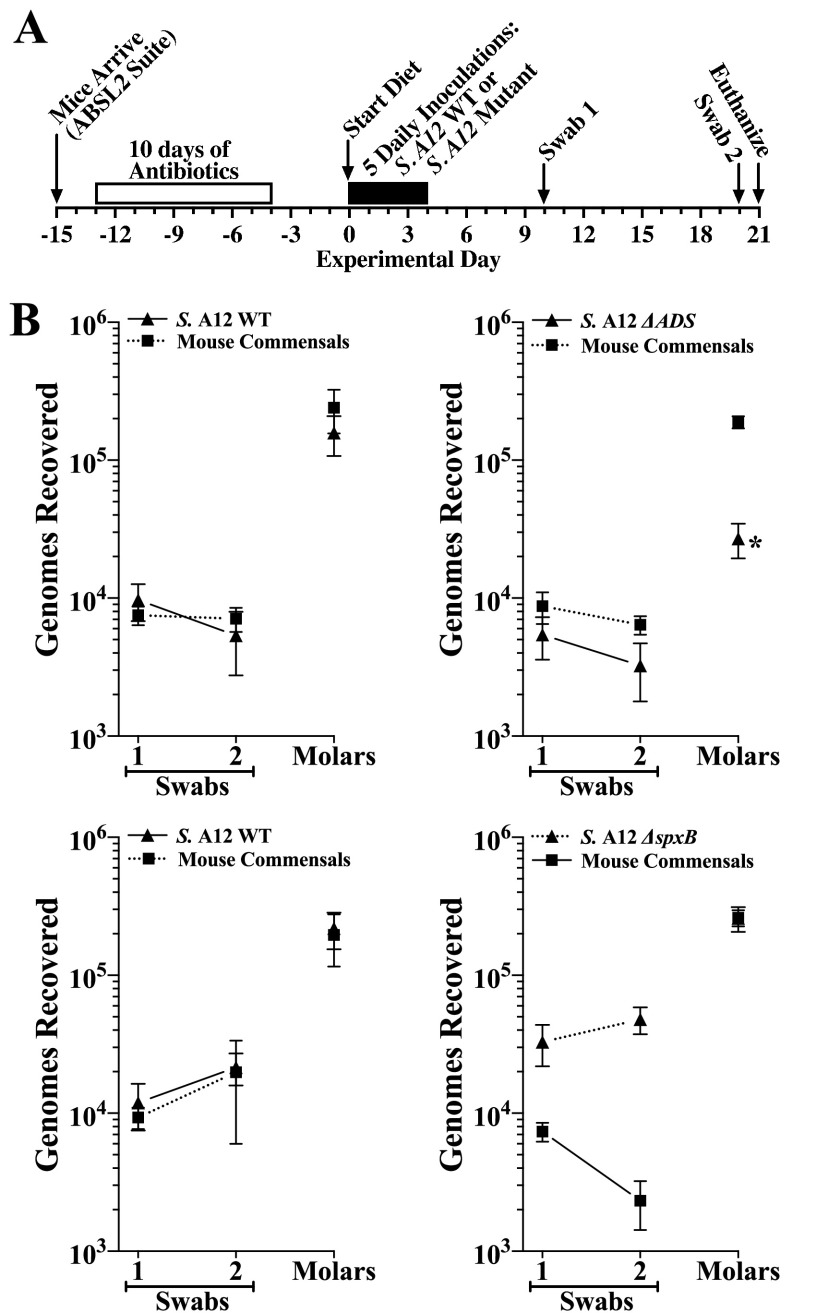
Comparison of colonization of the oral cavity and mandibular molars of mice by *Streptococcus* sp. A12 wild type (WT) or the indicated A12 mutant strain. (A) Timeline of key events in the experiment. (B) Colonization results from two separate experiments for each indicated inoculated strain and mouse oral commensals from oral swabs 1 and 2 taken at the times indicated in panel A and from sonicates of mandibular molars. Each experiment included a A12 WT control group. Mice were fed the high-sucrose diet plus 1.5% arginine with 4% sucrose water. Results are the mean ± SE (*n* = 10 mice per group) of recovered genomes estimated by qPCR. Statistical comparisons by one-way ANOVA with Tukey’s multiple-comparison test. *, *P* ≤ 0.05 versus A12 WT.

**Colonization.** Before testing A12 and its mutant strains, we first determined whether each mutant colonizes the mouse oral cavity and dentition. Ultimately, we wanted to examine A12 and its mutants under strongly cariogenic conditions to construct a rigorous test of each strain’s colonization and potential competitiveness against S. mutans, which would provide insights into the relative contribution of antagonism and pH moderation to competitive fitness. We therefore examined colonization with mice fed the high-sucrose diet with 1.5% arginine and 4% sucrose water. In subsequent competition experiments, added arginine was then posited to further amplify any deficits in competition associated with deletion of the ADS. In these initial colonization experiments, we used the same timeline for colonization as in the previous set of experiments with the 12 strains of human commensals (Fig. 2A). As shown in Fig. 2B, oral colonization by the ADS mutant was not significantly different than that of the A12 wild type (WT). However, dental colonization by the ADS-deficient mutant was approximately 80% less than that of the A12 WT, but still sufficient for further testing. In contrast, both oral and dental colonization of A12 Δ*spxB* were similar to those of the A12 WT. Oral and dental colonization of mouse commensals were not significantly different between the WT and each mutant group. The mean increase in body weights between groups was also not significantly different (Fig. S2B).

**Colonization by** **S. mutans**. In testing human commensals in competition experiments, our strategy was to first establish colonization by the commensal, followed by oral inoculations with S. mutans UA159 and monitoring of colonization. We reasoned that first establishing the human commensal likely reflects future clinical applications of probiotics in which patients at high caries risk would first undergo comprehensive removal of supragingival dental biofilms, followed by administration of a probiotic immediately thereafter and subsequent self-administration of probiotic at periodic intervals after brushing of teeth and/or use of an oral antiseptic. Because our initial oral colonization results indicated human oral commensals appeared to reach a steady state by experimental day 10 (swab 1) or earlier, we scheduled subsequent inoculations with S. mutans to start at experimental day 7. A concern with this strategy was that mice would not be inoculated with S. mutans until 6.5 to 7 weeks of age. It is established that colonization of rodents by S. mutans and subsequent development of dental caries is greatest when inoculated before weaning at age 21 days, when tooth eruption is in its early stages, and that colonization and caries then declines markedly with age (43, 53). In contrast, BALB/c mice older than 8 weeks of age were able to be colonized with S. mutans after antibiotic suppression of the oral microbiota (41, 42) and to induce measurable caries (54). It was thus necessary to first establish in our model how well S. mutans colonized and induced caries, but to also determine whether colonization and induction of caries by S. mutans decreased when inoculations were initiated at experimental day 7 compared to day 0. We used the high-sucrose diet with 4% sucrose water and extended the experiment to 7 weeks after the first inoculation with S. mutans to compare caries levels with those of prior experiments in which mice were challenged with S. mutans before weaning and fed a highly cariogenic diet for 7 weeks (43, 53).

**FIG 3:**
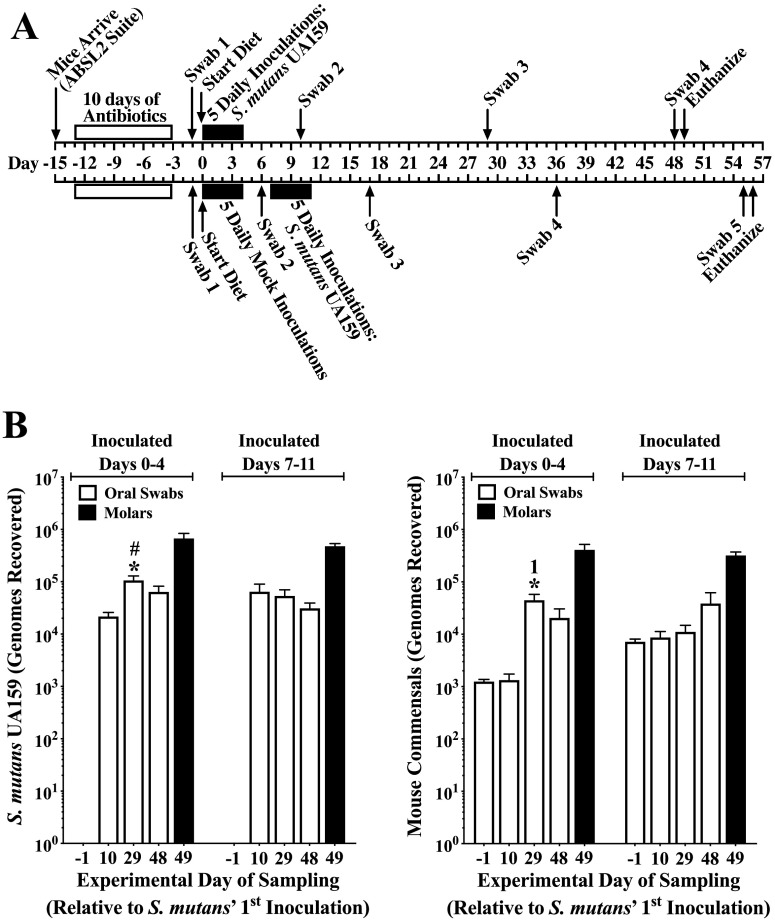
Comparison of S. mutans UA159 colonization when inoculated starting on day 0 versus day 7. (A) Timeline of major experimental events. (B) Colonization by S. mutans and mouse oral commensals from mandibular molars and from oral swabs at the indicated number of days relative to the first inoculation of S. mutans UA159, as determined by qPCR. Mice were fed the high-sucrose diet with 4% sucrose water. Results are from a single experiment and expressed as the mean ± SE of recovered genomes (*n* = 20 per group). *, *P* ≤ 0.05 versus the previous swab or an earlier swab as indicated by its number (e.g., 1 for swab 1). #, *P* ≤ 0.05 versus S. mutans swab day 48 of the day 7 group and mouse commensals in all swabs of the day 7 group and all swabs but day 29 of the day 0 group. Statistical comparisons by one-way ANOVA with Tukey’s multiple-comparison test.

The experimental timeline is shown in Fig. 3A. Mice were divided into two groups, with one group on experimental day 0 receiving the first of five daily inoculations of S. mutans, and the second group five daily inoculations without added bacteria (mock inoculations). On day 7, five daily inoculations were initiated. Figure 3B shows that oral colonization by S. mutans in both cases soon reached a steady state of slightly less than 10^5^ genomes, whereas murine autochthonous bacteria (mouse commensals) tended to increase more gradually, eventually reaching levels comparable to S. mutans. Molar colonization levels were statistically equivalent between S. mutans and murine autochthonous bacteria, both within and across the two conditions. Importantly, the incidence and severities of smooth surface and sulcal caries were highly similar after inoculating mice with S. mutans UA159 on experimental day 7 compared to day 0 (Table 2), demonstrating the absence of an age-related attenuation on colonization and induction of caries by S. mutans. Moreover, sulcal caries were comparable to those obtained in previous studies with mice inoculated prior to weaning and using Diet 2000 containing 56% sucrose with 5% sucrose water (43–45). There also were no differences in the mean increase in body weights between the two groups during the experiment (Fig. S2C).

**TABLE 2:** Development of caries and severities on molars of BalbC/J mice on the high-caries diet and inoculated with S. mutans UA159 on either experimental days 0 to 4 or experimental days 7 to 11 (with mock inoculations on days 0 to 4)^*a*^

Surface	Day 0	Day 7
Smooth surfaces		
Total E	3.42 (0.29)	3.80 (0.37)
Total Ds	1.79 (0.15)	2.10 (0.16)
Total Dm	1.21 (0.16)	1.00 (0.19)
Buccal E	0.32 (0.11)	0.50 (0.22)
Buccal Ds	0.00 (0.00)	0.05 (0.05)
Buccal Dm	0.00 (0.00)	0.00 (0.00)
Lingual E	2.10 (0.13)	2.55 (0.20)
Lingual Ds	1.79 (0.15)	2.05 (0.11)
Lingual Dm	1.21 (0.16)	1.00 (0.19)
Proximal E	1.00 (0.20)	0.75 (0.22)
Proximal Ds	0.00 (0.00)	0.00 (0.00)
Proximal Dm	0.00 (0.00)	0.00 (0.00)
Sulcal surfaces		
Total E	15.10 (0.75)	16.60 (0.60)
Total Ds	8.47 (0.93)	8.70 (0.62)
Total Dm	0.26 (0.10)	0.45 (0.13)

a Values are the means (SE) of Larson’s modified Keyes scores from a single experiment. Total smooth surface caries is the sum of buccal, lingual, and proximal caries. E, enamel affected; Ds, dentin exposed; Dm, 3/4 of the dentin affected. There were no differences (*P* > 0.10; *n* = 20) between day 7 and day 0 scores by ANOVA with Tukey’s multiple-comparison test.

**FIG 4:**
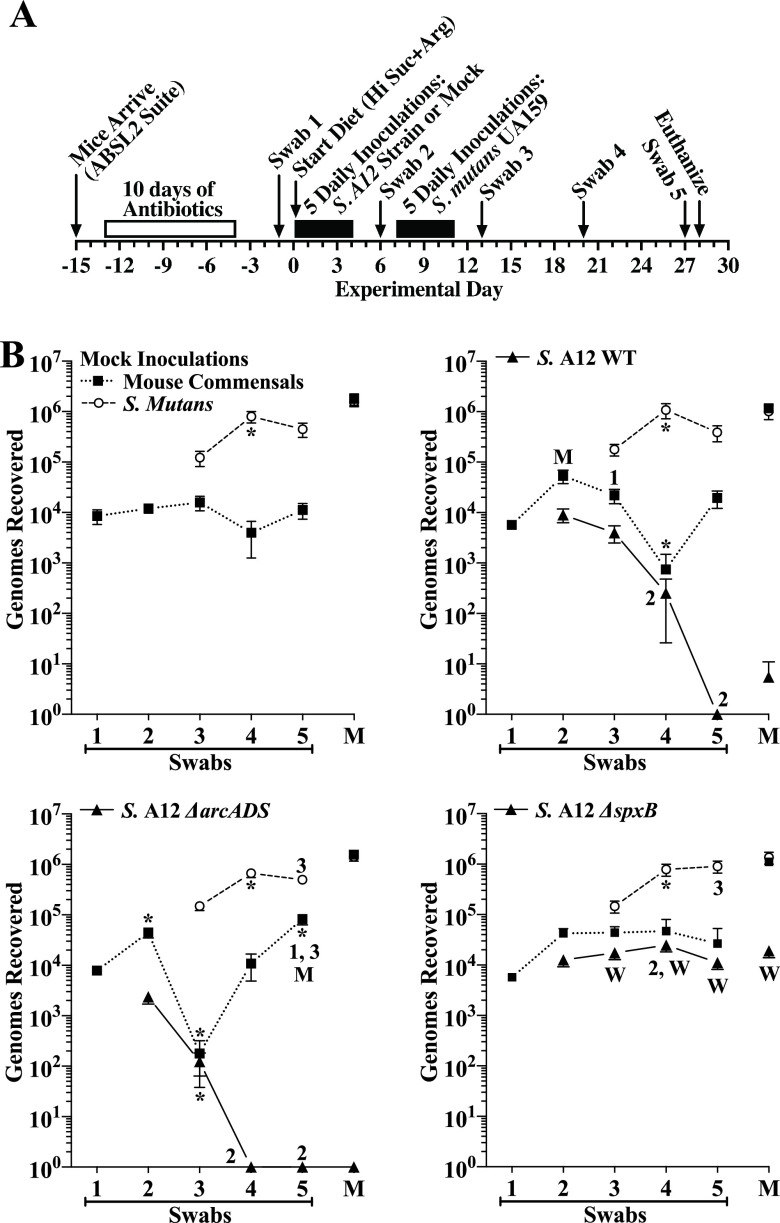
Comparison of colonization of the oral cavity and mandibular molars of mice by *Streptococcus* sp. A12 wild type (WT) or the indicated A12 mutant in competition with S. mutans UA159. (A) Timeline of key events in the experiment. (B) Results are from a single experiment that included each indicated inoculated A12 strain (filled triangles, solid lines), S. mutans UA159 (open circles, dashed lines) and mouse oral commensals (filled squares, dotted lines) from oral swabs 1 to 5 taken at the times indicated in panel A and from sonicates of mandibular molars (M). Mice were fed the high-sucrose diet plus 1.5% arginine with 4% sucrose water. Results are the mean ± SE (*n* = 14 mice per group) of recovered genomes estimated by qPCR. *, *P* ≤ 0.05 versus the previous swab or an earlier swab as indicated by its number (e.g., 2 for swab 2). M, *P* ≤ 0.05 versus the same point in the mock group. W, *P* ≤ 0.05 versus the A12 wild-type group. Statistical comparisons by one-way ANOVA with Tukey’s multiple-comparison test.

**Competition between** **S. mutans** **and A12.** Having established that A12 Δ*arcADS* and Δ*spxB* mutants colonize mice and further validating the competition experimental protocol, we next examined whether A12 altered colonization of S. mutans and/or the levels of autochthonous bacteria and if deletion of ADS activity or of pyruvate oxidase affected the outcomes. As shown in Fig. 4A, the major events in the experimental timeline are the same as in the competition validation experiment, except that the experiment was ended at experimental day 28, 21 days after starting S. mutans inoculations, but with more frequent oral swabbing to monitor oral colonization. As explained above, mice were fed the high-sucrose diet with 1.5% added arginine and 4% sucrose water. A group in which initial inoculations were without added bacteria (mock inoculations) was included as a control group for S. mutans alone. As shown in Fig. 4B, oral and dental colonization by S. mutans in the mock group were each robust, whereas oral colonization by murine commensals were lower but persistent. Similar to results of the validation experiment (Fig. 3B, inoculated days 7 to 11 group), dental colonization by mouse commensals was about 2 logs greater than its oral colonization and comparable to that of S. mutans. There were distinct differences in colonization between the three strains of A12. First, A12 WT was undetectable in swabs at experimental day 27 and barely detectable a day later in dental biofilms. A12 Δ*arcADS* appeared even less competitive against S. mutans, as oral colonization was undetectable a week earlier than the WT and was undetectable in molar biofilms. In stark contrast to A12 WT, oral colonization by A12 Δ*spxB* was unexpectantly persistent, with recoveries comparable to its recovery from dental biofilms. Oral colonization by murine autochthonous bacteria in this group was also persistent and similar to the mock group, while recoveries of mouse commensals in both the A12 WT and A12 Δ*arcADS* groups were erratic at times but nonetheless comparable or slightly higher than in the mock group by experimental day 27. Importantly, none of the A12 strains had an impact on dental colonization by S. mutans. Furthermore, the mean increase in body weights between the four groups was not significantly different (Fig. S2D).

### Examination of the competitiveness of four additional human commensal streptococci.

**FIG 5:**
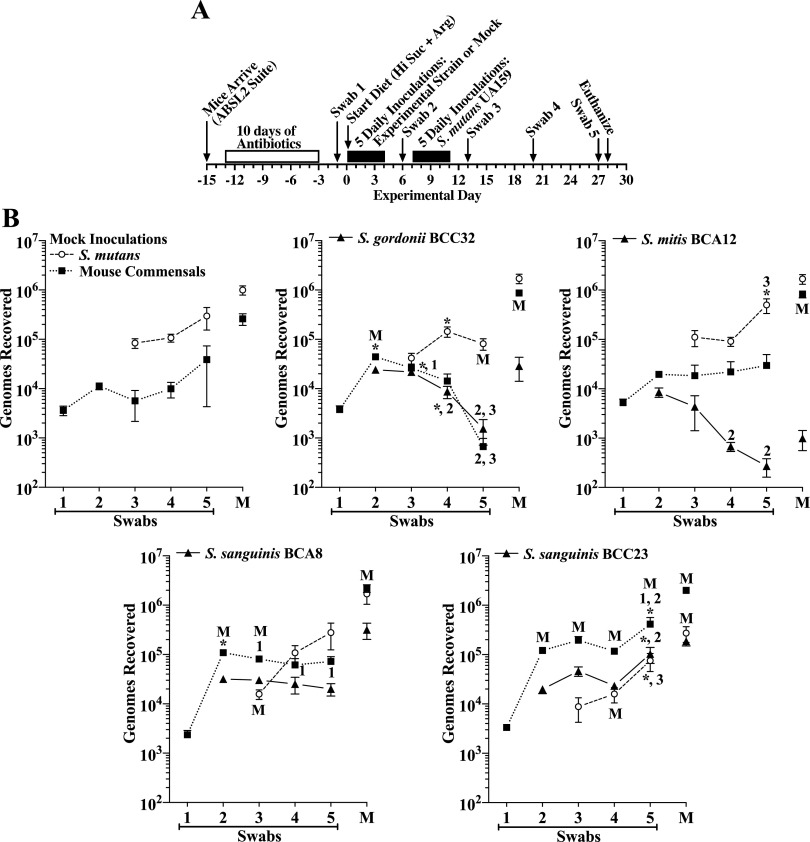
Comparison of colonization of the oral cavity and mandibular molars of mice by human oral commensal strains in competition with S. mutans UA159. (A) Timeline of key events in the experiment. (B) Results are from a single experiment that included each indicated inoculated human commensal strain (filled triangles, solid lines), S. mutans UA159 (open circles, dashed lines) and mouse oral commensals (filled squares, dotted lines) from oral swabs 1 to 5 taken at the times indicated in panel A and from sonicates of mandibular molars (M). Mice were fed the high-sucrose diet plus 1.5% arginine with 4% sucrose water. Results are the mean ± SE (*n* = 14 mice per group) of recovered genomes estimated by qPCR. *, *P* ≤ 0.05 versus the previous swab or an earlier swab as indicated by its number (e.g., 2 for swab 2). M, *P* ≤ 0.05 versus the same point in the mock group. Statistical comparisons by one-way ANOVA with Tukey’s multiple-comparison test.

We further assessed four additional human commensals in competition against S. mutans (i.e., S. gordonii BCC32, S. mitis BCA12, S. sanguinis BCA8, and S. sanguinis BCC23) because they exhibited the highest levels of colonization of dental biofilms under all four test diets (Fig. 1B). As a group, these strains also demonstrated various levels of ADS activity and antagonism (Fig. S1). Because an effective probiotic is expected to function well even when a patient is consuming a meal high in cariogenic carbohydrates, mice were given the high-sucrose diet with added arginine and provided 4% sucrose water. The experimental timeline (Fig. 5A) and inclusion of a mock group were the same as with the A12 strains. As shown in Fig. 5B, oral and dental colonization by S. mutans in the mock group were at high levels compared to murine autochthonous bacteria, although oral colonization of murine commensals was persistent.

Oral colonization by S. gordonii BCC32 progressively decreased to low levels, as did colonization by murine autochthonous bacteria. Notable, though, was the approximately 10-fold increase in oral colonization of mouse commensals following initial inoculations with S. gordonii BCC32, as well as the more than 3-fold decrease in S. mutans compared to the mock group at experimental day 27. A moderate level of S. gordonii BCC32 was recovered from dental biofilms. Compared to the mock group, the recovery from dental biofilms of S. mutans was unaltered, whereas autochthonous bacteria were enhanced. Importantly, the very low recovery (about 500 genomes) of mouse commensals from swabs on day 27 (swab 5) compared to nearly 10^6^ genomes recovered from mandibular molars on day 28, demonstrates that oral swabs capture bacteria primarily from nondental biofilms, most likely from saliva, epithelial biofilms (i.e., mucosal pellicles), and papillary groves of the tongue.

Oral colonization of S. mitis BCA12, like S. gordonii BCC32, progressively decreased with time. Conversely, oral colonization levels of murine autochthonous bacteria were consistent and levels of S. mutans increased 4-fold at experimental day 27. Dental colonization of S. mitis BCA12 was at a very low level, whereas dental colonization of S. mutans was unaltered, compared to the mock group. However, recovery of mouse commensals from dental biofilms was greater than in the mock group.

Unlike the two aforementioned strains, oral colonization by S. sanguinis BCA8 remained markedly consistent after introduction of S. mutans, and it populated molar biofilms at a level only about 4-fold lower than that of S. mutans. As for autochthonous bacteria, S. sanguinis BCA8 had a positive impact on dental colonization of this population and a transient positive effect on their oral colonization. Nevertheless, oral and dental colonization levels of S. mutans mimicked those of the mock group.

The most striking results were seen with S. sanguinis BCC23. Its oral colonization was stable and then increased during the final week. Its presence was associated with significant and consistently higher levels of murine autochthonous bacteria compared to the mock group. Conversely, the levels of oral colonization of S. mutans were depressed initially but increased during the final week in conjunction with S. sanguinis BCC23 and autochthonous bacteria. More importantly, dental colonization of S. mutans was nearly 4-fold lower than in the mock group and also markedly lower than autochthonous bacteria. Dental colonization by S. sanguinis BCC23 was greater than 10^5^ genomes, equivalent to that by S. mutans. Furthermore, mouse commensals in dental biofilms were significantly greater than in the mock group. There were also no differences in the mean increase in body weight between each of the five groups during the experiment (Fig. S2E).

### Assessment of caries.

Because S. sanguinis BCC23 effectively competed with S. mutans for colonization of molar biofilms, and all four groups enhanced dental colonization of autochthonous bacteria, we examined the mandibular molars of each of the groups to determine whether the incidence and severity of caries was impacted by a strain compared to the mock group. Bear in mind that this experiment was designed to examine colonization, ending only 3 weeks after the first inoculation with S. mutans, compared to 7 weeks in a typical caries experiment. Thus, caries levels were expected to be relatively low. Nonetheless, as shown in Table 3, mice colonized with S. sanguinis BCC23 demonstrated decreased severity of total smooth surface caries, due primarily to lower incidences in buccal and lingual lesions. Colonization by S. sanguinis BCA8 and S. gordonii BCC32 was associated with a trend toward fewer lesions on buccal surfaces and decreased severity of lingual caries, respectively. However, all four groups exhibited sulcal caries similar to the mock group.

**TABLE 3:** Development of caries and severities on mandibular molars of mice in the competition experiment^*a*^

Surface	Data for:				
	Mock infected	S. sanguinis BCC23	S. sanguinis BCA8	S. gordonii BCC32	S. mitis BCA12
Smooth surfaces					
Total E	3.74 (0.96)	1.86 (0.31)	3.00 (0.68)	2.64 (0.44)	3.36 (0.58)
Total Ds	1.07 (0.44)	0.14 (0.14)^*b*^	0.43 (0.20)	0.43 (0.17)	0.64 (0.23)
Total Dm	0.14 (0.10)	0.00 (0.00)	0.00 (0.00)	0.00 (0.00)	0.00 (0.00)
Buccal E	2.07 (0.69)	0.43 (0.17)^*b*^	1.71 (0.34)	0.93 (0.38)^*c*^	1.43 (0.51)
Buccal Ds	0.29 (0.29)	0.07 (0.07)	0.14 (0.10)	0.00 (0.00)	0.21 (0.21)
Buccal Dm	0.07 (0.07)	0.00 (0.00)	0.00 (0.00)	0.00 (0.00)	0.00 (0.00)
Lingual E	1.50 (0.23)	1.43 (0.20)	1.14 (0.33)	1.71 (0.19)	1.93 (0.20)
Lingual Ds	0.79 (0.24)	0.07 (0.07)^*b*^	0.29 (0.16)^*c*^	0.43 (0.17)	0.43 (0.14)
Lingual Dm	0.07 (0.07)	0.00 (0.00)	0.00 (0.00)	0.00 (0.00)	0.00 (0.00)
Proximal E	0.14 (0.14)	0.00 (0.00)	0.14 (0.14)	0.00 (0.00)	0.00 (0.00)
Proximal Ds	0.00 (0.00)	0.00 (0.00)	0.00 (0.00)	0.00 (0.00)	0.00 (0.00)
Proximal Dm	0.00 (0.00)	0.00 (0.00)	0.00 (0.00)	0.00 (0.00)	0.00 (0.00)
Sulcal surfaces					
Total E	7.86 (1.40)	8.00 (0.56)	8.93 (0.77)	5.86 (0.90)	8.00 (0.97)
Total Ds	1.50 (0.53)	0.57 (0.17)	1.36 (0.41)	1.00 (0.38)	1.57 (0.40)
Total Dm	0.14 (0.10)	0.00 (0.00)	0.07 (0.07)	0.07 (0.07)	0.07 (0.07)

a Values are the means (SE) of caries and severities. E, enamel affected; Ds, dentin exposed; Dm, 3/4 of the dentin affected. Comparisons by ANOVA with Tukey’s multiple-comparison test. *n* = 14 for each group.

b *P* ≤ 0.05 versus mock.

c *P* ≤ 0.10 versus mock.

### Reproducibility between experiments.

**FIG 6:**
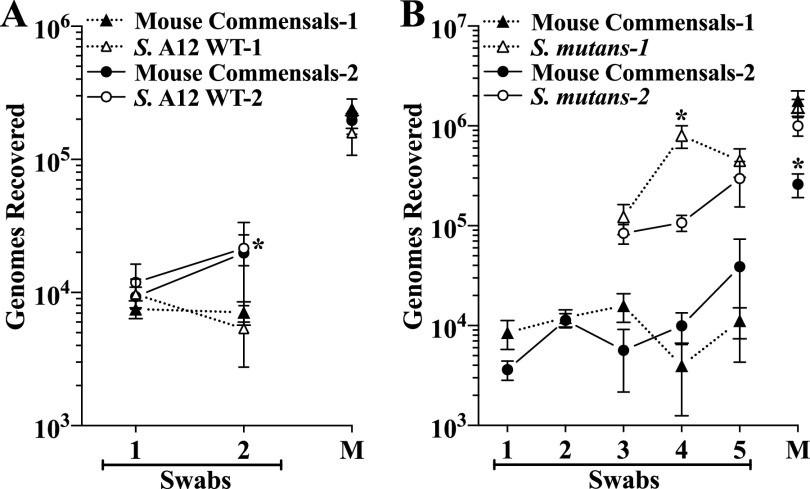
Comparisons between separate identical experiments of colonization of the oral cavity and mandibular molars of mice. (A) Comparisons between the two experiments (1 and 2) in Fig. 2B of colonization by A12 wild-type (WT) and murine autochthonous commensals (mouse commensals). (B) Comparisons between the two mock groups in competition experiments of Fig. 4B (1) and Fig. 5B (2) that included colonization by S. mutans and mouse commensals. Results are the mean ± SE (panel A, *n* = 10 mice per group; panel B, *n* = 14) of recovered genomes estimated by qPCR. Statistical comparisons between swabs at a given point are by one-way ANOVA with Sidak’s multiple-comparison test. Statistical comparisons between recoveries from mandibles are by the two-tailed unpaired *t* test. *, *P* ≤ 0.05 versus the same point in the alternate experiment.

There were two cases in which experimental conditions were repeated in separate experiments, providing an opportunity to assess the reproducibility of bacterial recoveries in the *in vivo* model. The first case was in two experiments testing colonization by WT A12, in which there was only a single significant difference, a 4.0-fold difference in the recovery of A12 at swab 2 (Fig. 6A). The second case included the two mock groups in competition experiments with S. mutans. There are two instances of significant differences between these two experiments, recovery of S. mutans at swab 4 (7.5-fold) and recovery of mouse commensals from mandibular molars (6.9-fold) (Fig. 6B). Overall, though, there was good reproducibility between experiments.

## DISCUSSION

### Diet and colonization by human commensal streptococci and autochthonous bacteria.

**Oral and dental colonization in relation to dietary sucrose.** In the context of dental caries, dietary carbohydrates are a critical determinant of the oral microbiome and oral health, due in large part to the ability of S. mutans to rapidly utilize sucrose to produce a structural matrix of insoluble glucans that can greatly enhance the cariogenic potential of oral biofilms (1). The high cariogenicity of the high-sucrose diet used in this study was confirmed, as smooth surface and sulcal caries of mice inoculated with S. mutans alone were comparable to prior experiments that incorporated Diet 2000, containing 56% sucrose and 5% sucrose water (43). In contrast, the average diet contained nearly 70% less sucrose, and mice were supplied with sterile drinking water without added sucrose. Omission of sucrose from the drinking water further reduces the cariogenicity of a diet, likely by decreasing the frequency of exposure of S. mutans to sucrose. For example, in a pilot study using our earlier mouse caries model with mice fed Diet 2000, the incidences of total smooth surface caries, total sulcal caries, and recovery of S. mutans from molar sonicates were 84%, 62%, and 49% less, respectively, in mice provided sterile water than mice provided with 5% sucrose water (D. J. Culp, unpublished observations). Therefore, in light of the differences in cariogenicity between the average and high-sucrose diets, the ability of the great majority of candidate probiotics to colonize the oral cavity and dental biofilms at relatively moderate to high levels, regardless of the level of dietary sucrose, is considered a reflection of their adaptation to dental biofilms of caries-free individuals and is a highly desirable attribute, as diet will likely vary in cariogenicity among patients taking probiotics. In addition, colonization of nondental sites in the oral cavity, such as oral epithelium and within papillary groves of the tongue, potentially creates a reservoir for persistent recolonization of dental biofilms. Of particular note was the inexplicable extreme increase in molar colonization by S. intermedius A3 with the high-sucrose diet compared to the average diet. Additional investigations are required to explain this phenotype.

### Added dietary arginine and colonization.

Added dietary arginine had no consistent positive impact on either oral or dental colonization by nine strains and was associated with decreased dental colonization of two strains. The absence of a positive impact suggests those strains with relatively high ADS activity (e.g., S. gordonii BCC32, S. sanguinis BCC23, and A12) are sufficiently aciduric or do not require increased ADS activity because base production via urease from salivary urea combined with the ADS utilizing arginine supplied via salivary peptides and dietary proteins, is enough to counteract any acids produced by the autochthonous bacterial population. Conversely, colonizing strains not impacted by arginine, but with very low ADS activity (e.g., S. sanguinis BCA8, S. mitis BCA12, and S. mitis BCC08), may be sufficiently aciduric. Interestingly, S. intermedius A3 with very high ADS activity was negatively impacted by arginine. Added arginine may have increased environmental pH to levels substantially above neutrality (e.g., 8.0), levels where many human streptococci display poor growth (10), thus explaining the extreme decrease in molar colonization by both S. intermedius A3 and murine autochthonous bacteria with added dietary arginine.

### Murine autochthonous oral bacteria.

Murine autochthonous oral bacteria recovered rapidly after antibiotic treatment as observed in each mock inoculation group of the two competitive colonization experiments, where recoveries of mouse commensals on day –1 were no more than 2-fold lower than on day 6. Furthermore, the inability of *S. cristatus* A52 to colonize mice demonstrates that inoculated strains must compete with murine autochthonous bacteria. Similar levels of either oral or molar colonization by autochthonous bacteria across the four diets indicates that added sucrose or arginine do not alone impact the total population of mouse commensals. The presence of a significant population of mouse resident flora upon introduction of human oral commensal strains, combined with establishment of 11 of the 12 strains under different dietary conditions, again speaks to the adaptation of these strains to the oral environment.

### Examining specific strains and isogenic mutants in competition against S. mutans.

**A12 and ADS in colonization and competition.** The poor competitiveness of A12 WT against S. mutans *in vivo* was unanticipated. Of the 12 strains, A12 has very high ADS activity and the highest level of antagonism *in vitro* and was recently shown to have multiple strategies to antagonize S. mutans, such as inhibition of *comX*-inducing peptide signaling (5). Nevertheless, the more rapid decrease in oral colonization by A12 Δ*arcADS* versus the WT when in competition with S. mutans may be due to decreased competitiveness against S. mutans, as the mutant displayed similar oral colonization as A12 WT in the absence of S. mutans. In contrast, molar colonization by A12 Δ*arcADS* in the absence of S. mutans was significantly less than that of the WT, indicating that ADS contributes to A12’s competitiveness against autochthonous bacteria in dental biofilms under cariogenic conditions. Perhaps one or more members of the population of mouse commensals are sufficiently acidogenic that A12’s ADS is required to help support dental colonization. Although A12 Δ*arcADS* failed to colonize molar biofilms in the presence of S. mutans, it is unclear whether ADS activity exerts any additional competitiveness for A12 WT against S. mutans in addition to competitiveness against autochthonous bacteria.

### A12 pyruvate oxidase.

Surprisingly, A12 Δ*spxB* persisted orally at levels significantly greater than A12 WT but did not interfere with oral and dental colonization by S. mutans. In a dual-species biofilm model of A12 and S. mutans, both species formed microcolonies adjacent to each other without any detectable integration, with a decrease in both biomass and biofilm maximal thickness compared to biofilms of S. mutans alone (52). It would thus be interesting to determine in dental biofilms *in vivo* whether A12 Δ*spxB* is localized adjacent to S. mutans and if the ADS is required for its dental and persistent oral colonization.

A closer look at the combined results of all three strains of A12 provides a putative explanation for persistent oral colonization of A12 Δ*spxB.* First, the total population of mouse oral commensals was not impacted when S. mutans was introduced in the mock group, suggesting autochthonous bacteria readily adapt to introduction of S. mutans. However, addition of an A12 strain expressing pyruvate oxidase activity (i.e., A12 WT and A12 Δ*arcADS*) appears to initiate microbial interactions that are detrimental to A12 and negatively affect autochthonous bacteria but allow the commensal population to eventually recover. A similar scenario may also explain the poor colonization of dental biofilms by A12. Additional studies are thus warranted to determine how production of H_2_O_2_ by A12 alters competitive mechanisms of oral autochthonous commensals when S. mutans is entered into the environment. In a broader context, the results call for more thorough investigation of the influence of H_2_O_2_ production by commensal streptococci on the establishment, persistence, and virulence of oral pathogens and pathobionts (55).

### Interrogation of four additional human commensal streptococci.

 Interestingly, the total population of mouse commensals within dental biofilms when mice were challenged with S. mutans was enhanced in the presence of each of the four strains, indicating that these strains promote the resilience and competitiveness of dental biofilm residents. Further investigations of cooperative mechanisms beneficial to autochthonous bacteria may elucidate additional approaches to help prevent caries. With respect to S. gordonii BCC32, its inability to affect dental colonization by S. mutans is consistent with previous results in rats with S. gordonii Challis CH1, which failed to alter molar colonization by S. mutans or the incidence of caries, despite showing antagonism against S. mutans *in vitro* (56). Only S. sanguinis BCC23 and S. sanguinis BCA8 demonstrated persistent oral colonization and were recovered at high levels from dental biofilms. More importantly, S. sanguinis BCC23 attenuated S. mutans colonization of molar biofilms and showed promise in lowering smooth surface caries. Interestingly, colonization by S. sanguinis of newly acquired teeth in children precedes and also delays colonization by S. mutans, suggesting an inhibitory effect on S. mutans (57). Furthermore, within cavitated lesions, in which S. mutans accounts for up to 55% of the microbiota, S. sanguinis persists (4). Thus, other oral strains of S. sanguinis may represent an important source of probiotic strains. BCC23 is only the second oral commensal strain with demonstrated competitiveness against S. mutans under such highly cariogenic conditions *in vivo* (58). Limited clinical studies of potential probiotics incorporating oral streptococci have reported lower caries development in children (27) and decreased levels of S. mutans in either saliva (25, 26) or dental plaque (59), demonstrating the potential for effective anticaries probiotics based on strains of oral commensal streptococci. S. sanguinis BCC23 is therefore a highly attractive probiotic candidate and can serve as an important tool to elucidate competitive mechanisms that hinder the establishment, persistence, and virulence of S. mutans. For example, because S. sanguinis BCC23 has relatively high ADS activity, it would be of interest to determine the effects on competitiveness of added dietary arginine and of deletion of ADS activity.

### Antagonism *in vitro* does not correlate with competitiveness *in vivo*.

A closer look at antagonism *in vitro* of all five strains examined for competitiveness against S. mutans shows that antagonism levels of A12 and S. gordonii BCC32 are about 40-fold and 2-fold greater, respectively, than that for S. sanguinis BCC23. S. mitis BCA12 and S. sanguinis BCA8 have only slightly lower levels than S. sanguinis BCC23 (see Fig. S3). Furthermore, the failure of strains other than S. sanguinis BCC23 to affect dental colonization by S. mutans is not related to their ability to colonize dental biofilms, as each strain colonized molar teeth at levels higher or slightly lower than S. sanguinis BCC23 when mice were fed the same diet (Fig. S3). These combined results strongly suggest antagonism against S. mutans, *in vitro*, which is highly dependent on growth conditions, does not correlate with competitiveness *in vivo*, at least under the conditions referenced here, and further warrants that caution should be exercised when extrapolating *in vitro* phenotypes to the *in vivo* environment. *In vivo* testing of competitiveness of a candidate probiotic thus represents an important discriminating assessment to identify strains for further study, especially in light that the phenotypic heterogeneity displayed by oral streptococci is not species specific, nor always reflected by genotype (18). Initial identification in an animal model of strains such as S. sanguinis BCC23 that colonize, persist, and compete against S. mutans can further reduce unnecessary clinical studies that test strains based solely on *in vitro* data. However, because functional genomics of commensal strains such as A12 are identifying previously unrecognized genetic elements that function in competitiveness *in vitro*, genes shown to impact competitive fitness may eventually serve as biomarkers to better recognize beneficial organisms *in vivo* (5). Such efforts will nevertheless require *in vivo* examination of isogenic mutants.

Collective results demonstrate that human oral commensals strongly associated with dental health are generally well adapted to colonize both the soft and hard tissues of mice under highly cariogenic and healthier dietary conditions, and they identify a highly attractive probiotic candidate, S. sanguinis BCC23. Health-associated dental isolates from humans thus represent a source of putative probiotic strains with the potential to colonize dental and oral biofilms of patients, regardless of diet. Results further demonstrate that the *in vivo* model is sensitive and reproducible, representing a reliable platform to rigorously test putative probiotic strains to colonize soft and hard tissues, to compete against severe challenge with a highly virulent pathogen, to support autochthonous commensals, and to reduce the incidence of caries. The model is also amenable to interrogations of key molecular mechanisms responsible for competitiveness against S. mutans and persistent colonization of epithelial and/or dental biofilms. The model is further amenable to exploring the effectiveness of the dose and frequency of administration of a probiotic or prebiotic. We therefore demonstrate the utility of *in vivo* assessments to more stringently evaluate the oral fitness of candidate strains to help facilitate the rational design and optimization of novel probiotic strategies to target microbial ecology in protection of supragingival dental surfaces.

## MATERIALS AND METHODS

### Procedures with mice.

The mouse model is a modification of a previously described mouse caries model (44). All procedures with solutions and samples were performed under biosafety level 2 (BSL2) conditions, and mice were kept under Animal Biosafety Level 2 (ABSL2) conditions. Briefly, inbred 3-week-old female specific-pathogen-free (SPF) BALB/cJ mice (The Jackson Laboratory, Bar Harbor, ME) were placed in pairs in sterile cages. Two days later, mice were given drinking water containing 0.8 mg/ml sulfamethoxazole/0.16 mg/ml trimethoprim for a total of 10 days to suppress indigenous oral bacteria, followed by a 3-day washout period with sterile drinking water. On the following day (designated experimental day 0) mice were placed on one of four diets (see Results) and inoculated daily for 5 successive days with 50 µl of 1.5% (wt/vol) carboxymethylcellulose in saliva buffer (50 mM KCl, 1.0 mM KPO_4_, 0.35 mM K_2_HPO_4_, 1.0 mM CaCl_2_·2H_2_O, 0.1 mM MgCl_2_·6H_2_O, pH 6.5) containing approximately 1 × 10^9^ cells of the indicated strain grown to an optical density at 600 nm (OD_600_) of 0.55 to 0.70 or inoculated with 1.5% (wt/vol) carboxymethylcellulose alone (mock inoculations). In competition experiments, mice also underwent on experimental day 7 five consecutive daily inoculations with approximately 1 × 10^9^ cells of S. mutans UA159. At the end of each experiment, mice were euthanized by CO_2_ asphyxiation followed by cervical dislocation. The protocol was reviewed and approved by the Institutional Animal Care and Use Committee at the University of Florida (IACUC protocol 201810470). More detailed information regarding the caging and feeding of mice and the preparation and delivery of inoculants is given in the supplemental material, page 8.

### Bacterial strains and growth conditions.

Low-passage S. mutans UA159 and human commensal streptococcal isolates were initially grown in BHI broth (brain heart infusion broth powder, 3.7%, containing 0.2% glucose; Difco Laboratories, Detroit, MI) at 37°C in a 5% CO_2_ aerobic environment to an optical density at 600 nm (OD_600_) of 0.5. Then glycerol was added to 25% (vol/vol), and 40 aliquots of 1 ml were frozen at –75°C. For A12 mutants, the BHI contained 1 mg/ml kanamycin. Each frozen aliquot was used either to grow cells in BHI for isolation of genomic DNA for qPCR standards or to prepare a single inoculant.

### Oral swabs.

Oral swabs were taken at indicated intervals using HydraFlock 6-inch sterile micro ultrafine flock swabs (Puritan Medical Products, Guilford, ME). Swab tips were vortexed (3 times for 5 s) in 1 ml sterile phosphate-buffered saline (PBS), the tips were removed, and 200 µl of ice-cold PBS containing approximately 5 × 10^8^ depurinated cells of laboratory strain S. mitis UF2 (see below) was added. The tube was then vortexed for 5 s and centrifuged (10,000 × *g*, 10 min at 4°C) to pellet recovered cells. Cell pellets were then processed for DNA isolation using the DNeasy UltraClean microbial kit (Qiagen Inc., Germantown, MD) as per the manufacturer’s instructions. More detailed information regarding swabbing of mice and recovery of bacteria is given in the supplemental material.

### Preparation of depurinated cells.

In preliminary experiments, employment of a high concentration of depurinated cells was found to greatly enhance quantitative pelleting and recovery of low cell numbers and subsequent DNA, thus increasing the sensitivity of qPCR assays. The cells walls of depurinated cells remain intact and therefore at high concentrations act as a carrier to help limit nonspecific binding and promote pelleting of recovered bacteria. Purine bases in genomic DNA are lost by depurination, producing apurinic sites and rendering DNA undetectable in all qPCR assays. To prepare depurinated cells, a 200 ml culture (OD_600_, 0.5) of laboratory strain S. mitis UF2 in BHI was pelleted (4 × 50 ml at 10,000 × *g* for 7 min at 4°C), and each pellet was resuspended in 11 ml sterile ice-cold PBS. Cells were pooled and centrifuged again. The pellet was resuspended in 35 ml of 0.2 N HCl and placed in a 70°C water bath for 90 min with vortexing (5 × 2 s) every 15 min. Cells were then pelleted as before, and the 90-min incubation in fresh 0.2 N HCl was repeated. Cells were washed 3 times with 30 ml sterile ice-cold PBS. Before the third centrifugation, the cell concentration was estimated from the OD_600_, and the subsequent cell pellet was resuspended in sterile ice-cold PBS to a concentration of approximately 2.5 × 10^9^ cells/ml and then aliquoted and stored at –75°C.

### Dental colonization.

To assess dental colonization, the left and right halves of each mandible were aseptically extracted by first breaking the fibrous symphysis at the rostral midline, then gripping one incisor and pulling the left or right half of mandibular bone away from the temporal mandibular joint and nearly all associated soft tissue. Then, under a dissecting microscope, any remaining extraneous soft tissue near the molar teeth was removed by scraping with a scalpel followed by removal of bone approximately 2 mm anterior and posterior to the three molar teeth. Molar teeth with remaining underlying bone were sonicated on ice in 1 ml sterile PBS, pH 7.4, in siliconized 2-ml microcentrifuge tubes. Molar teeth with remaining bone were then aseptically removed using sterile forceps. Approximately 5 × 10^8^ depurinated cells of laboratory strain S. mitis UF2 were then added, and the tube was vortexed for 5 sec and centrifuged (10,000 × *g*, 10 min at 4°C). Cell pellets were then processed for DNA isolation as described above for swabs. More detailed information regarding recovery of bacteria from mandibular molars is given in the supplemental material.

### Quantitative PCR.

Quantitative PCR was used to estimate total recovered bacterial genomes and recovered genomes of inoculated strains in each DNA sample. DNA isolation using the DNeasy UltraClean microbial kit (Qiagen) resulted in 50 µl of DNA that was diluted to 125 µl with nuclease-free water, resulting in DNA in 4 mM Tris-HCl, pH 8.0. Samples from swabs were stored at –75°C in aliquots. Samples from molars were treated similarly but further diluted 10-fold with 4 mM Tris-HCl, pH 8.0, to eliminate interference in qPCR assays caused by unknown components in the samples and then aliquoted and stored at –75°C. Each qPCR assay included 9 µl of diluted DNA. The resultant genome numbers from the average of triplicates were then multiplied by either 13.89 (for swab DNA) or 138.89 (for mandibular DNA) to calculate the total recovered genomes in each sample. To estimate the total recovered bacteria, degenerate primers were used to PCR amplify conserved regions of the ubiquitous single-copy gene, *rpsL* (30S ribosomal protein S12) (48). Recovery of mouse commensals was then estimated by subtracting the recovered genomes of inoculated strains from the total recovered bacterial genomes. The primers and qPCR conditions used are given in Table S1. Standard curves were derived from DNA samples isolated from each strain grown to mid-exponential phase in BHI. S. mutans UA159 was used as the standard for *rpsL* assays. Efficiencies, slopes, and *r*^2^ values for standard curves were greater than 90%, –3.205, and 0.978, respectively. Statistical comparisons of colonization between groups were by one-way analysis of variance (ANOVA) with Tukey’s multiple-comparison test. More detailed information regarding development of qPCR assays is given in the supplemental material.

### Caries scoring.

Smooth surface and sulcal caries of mandibular and maxillary molars were scored by a single calibrated examiner using Larson’s modification of the Keyes scoring system, as described previously (44). The linear evaluations of carious enamel involvement are expressed as E, while severities of carious lesions, based on degree of dentin involvement, are expressed as Ds (dentin exposed) and Dm (3/4 of the dentin affected). To stabilize variances, caries scores were expressed as proportions of their maximum possible values (124 for smooth surface caries and 56 for sulcal surface caries), and then the arcsine of the square root of the proportions was calculated, as described previously (43). Transformed scores were compared by analysis of variance with Tukey’s multiple-comparison test using Prism v8.1 (GraphPad Software, San Diego, CA). More detailed information regarding preparing jaws for caries scoring is given in the supplemental material.

### Construction of A12 mutant strains.

The construction and characterization of A12 Δ*spxB* was described previously (9). The mutant strain, A12 Δ*arcADS*, was constructed in a similar manner by double-crossover recombination using linear DNA assembled through a Gibson assembly kit (New England BioLabs, Beverly, MA). Briefly, primers were designed to PCR amplify two DNA fragments flanking the coding sequences of five genes within the ADS operon from the 5′ end of *arcA* to the 3′ end of *arcT* and containing at least 27 bases of sequence that overlapped with the termini of the nonpolar resistance cassette in pALH124 (60) (see Fig. S4). The two flanking DNA fragments and the kanamycin resistance cassette were mixed in equimolar concentrations in a single isothermal ligation reaction. Overnight cultures of A12 were inoculated into fresh BHI cultures, and 0.5 µg of the ligated DNA products was used to transform A12 in BHI using 50 nM A12 synthetic competence-stimulating peptide of S. mutans to induce competence. After 3 h of incubation, cells were plated onto BHI agar with 1 mg/ml kanamycin, and isolated colonies were picked for PCR verification. PCR products from positive transformants underwent DNA sequencing to ensure correct insertion and the absence of mutations in the flanking regions used for homologous recombination. Primer sequences are listed in Table S3.

### ADS activity.

A12 Δ*arcADS* was confirmed for the inability to express ADS by monitoring citrulline production from arginine using protocols detailed previously (7). Briefly, mid-log-phase cultures in tryptone-yeast (TY) medium containing 25 mM galactose, with or without 10 mM arginine, were permeabilized using toluene-acetone. An aliquot was assessed for protein using the Pierce (Waltham, MA, USA) bicinchoninic acid protein assay kit, and then ADS activity was determined and normalized to protein. Assays were performed in biological duplicates and repeated two independent times with wild-type A12 as the positive control.

## Supplementary Material

Supplemental file 1
