# Supplementary material for: In Vivo Colonization with Candidate Oral Probiotics Attenuates Streptococcus mutans Colonization and Virulence
Source: Appl Environ Microbiol. 2021 Jan 29;87(4):e02490-20. doi: 10.1128/AEM.02490-20 (PMC7851695; doi:10.1128/AEM.02490-20)
Supplement: Supplemental file 1 [file AEM.02490-20-s0001.pdf]

Supplemental Material

In Vivo Colonization with Candidate Oral Probiotics Attenuates Colonization and Virulence of *Streptococcus mutans*

David. J. Culp, William Hull, Matthew J. Bremgartner,
Todd A. Atherly, Kacey B. Christian, Mary Killeen, Madeline R. Dupuis,
Alexander C. Schultz, Brinta Chakraborty, Kyulim Lee, Deneen S. Wang,
Verisha Afzal, Timmy Chen and Robert A. Burne

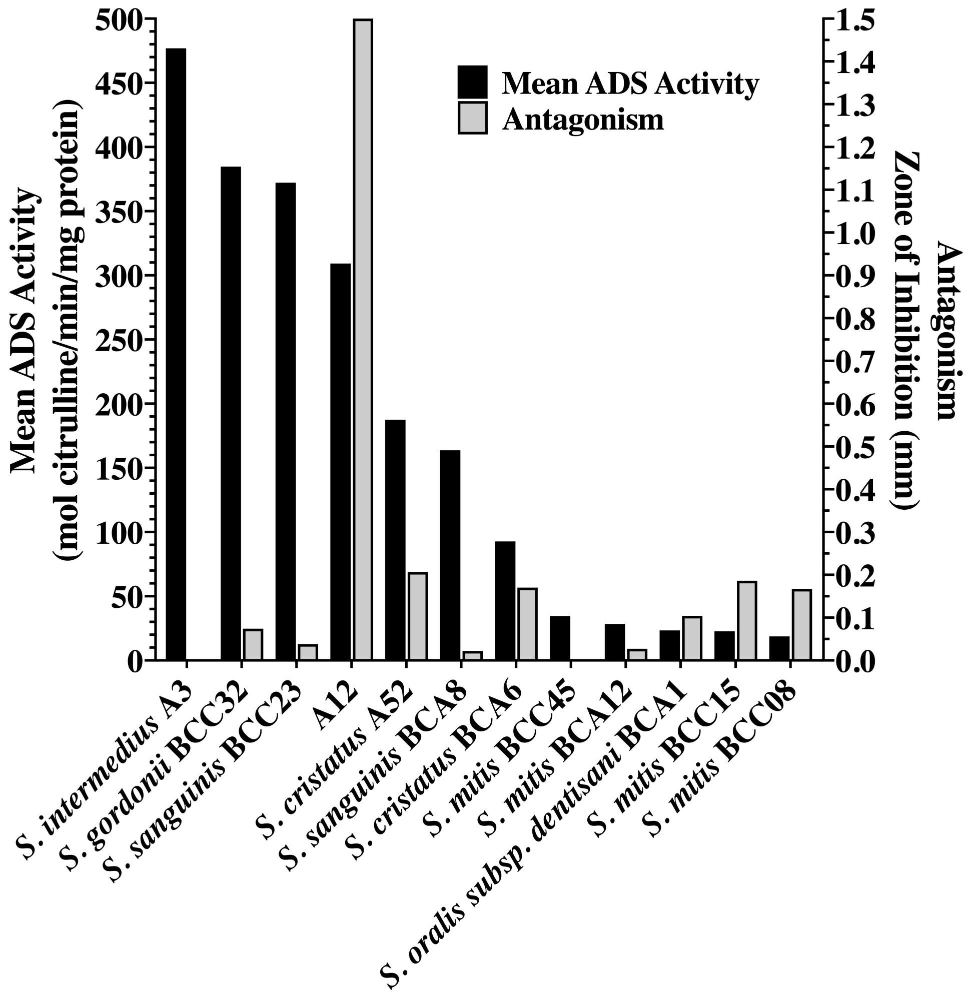

Fig. S1. ADS and antagonism against *Streptococcus mutans* UA159 by selected human commensal streptococci as characterized previously (1). Each strain was tested for generation of citrulline from arginine via the arginine deiminase system (ADS). Antagonism was assessed by measurement of the zone of inhibition in competition between commensal streptococci and *S. mutans* UA159 on BHI agar plates after first spotting the commensal followed the adjacent spotting of *S. mutans* UA159 24 h later.

1. Velsko, I. M., B. Chakraborty, M. M. Nascimento, R. A. Burne and V. P. Richards (2018). "Species Designations Belie Phenotypic and Genotypic Heterogeneity in Oral Streptococci." mSystems 3(6).

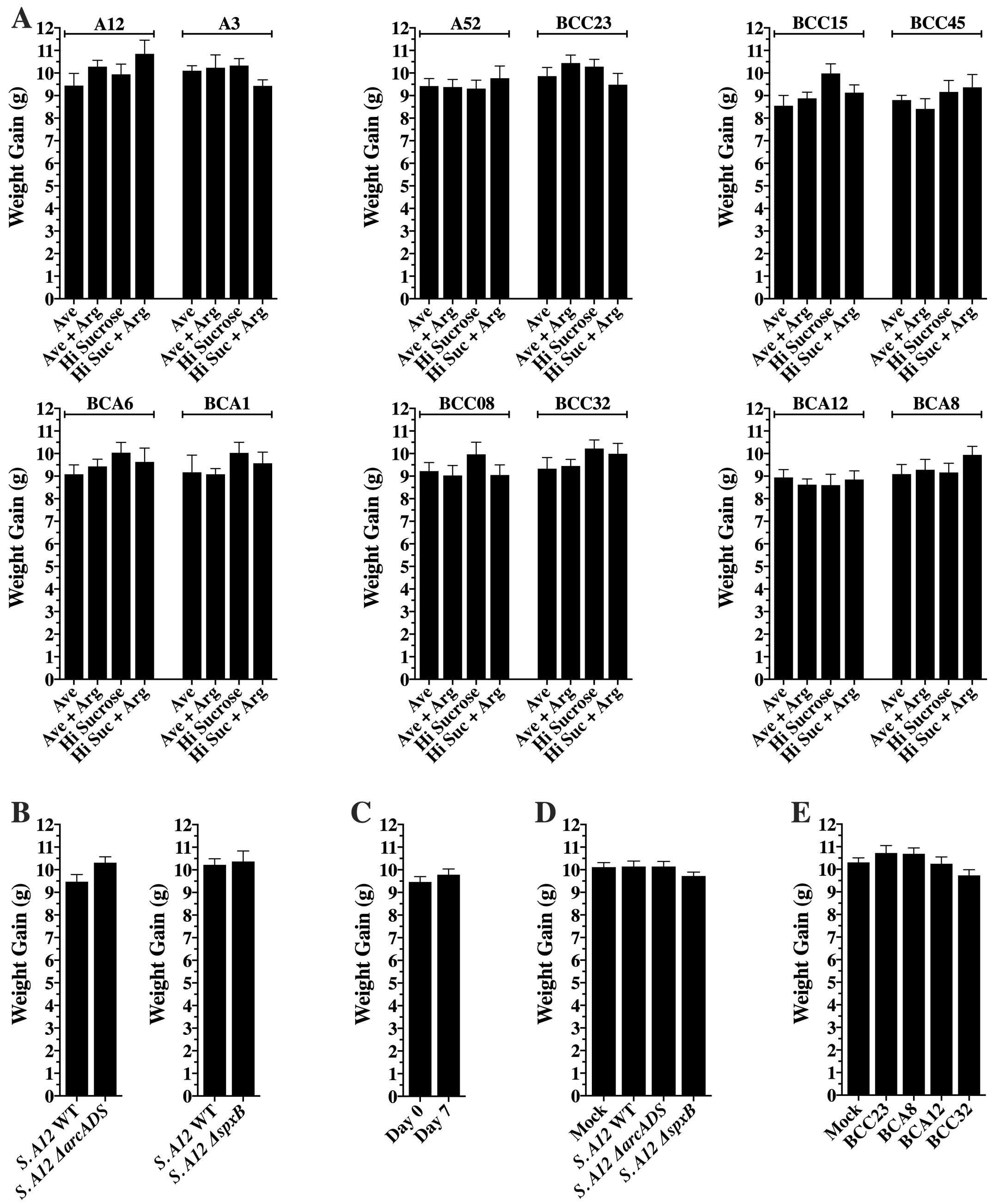

Fig. S2. Weight gains of mice during each experiment. **A.** Weight gains of mice during each of the six initial experiments examining colonization of two human commensal strains of streptococci when fed each test diet. **B.** Weight gains of mice during two experiments comparing colonization of A12 WT and mutant strains. **C.** Weight gains of mice inoculated with *S. mutans* UA159 on day 0 versus day 7. **D.** Weight gains of mice during competition experiments of A12 WT and mutant strains with *S. mutans* UA159. **E.** Weight gains of mice during competition experiments of human commensal strains of streptococci with *S. mutans* UA159. All values are mean ± SE with n for each group = 10, A and B; 20, C;14, D and E. In all experiments $p > 0.05$ in comparisons among all groups in an experiment by one-way ANOVA with the Tukey-Kramer post hoc test (A, D and E) or Student's t test, unpaired, two-tailed (B and C).

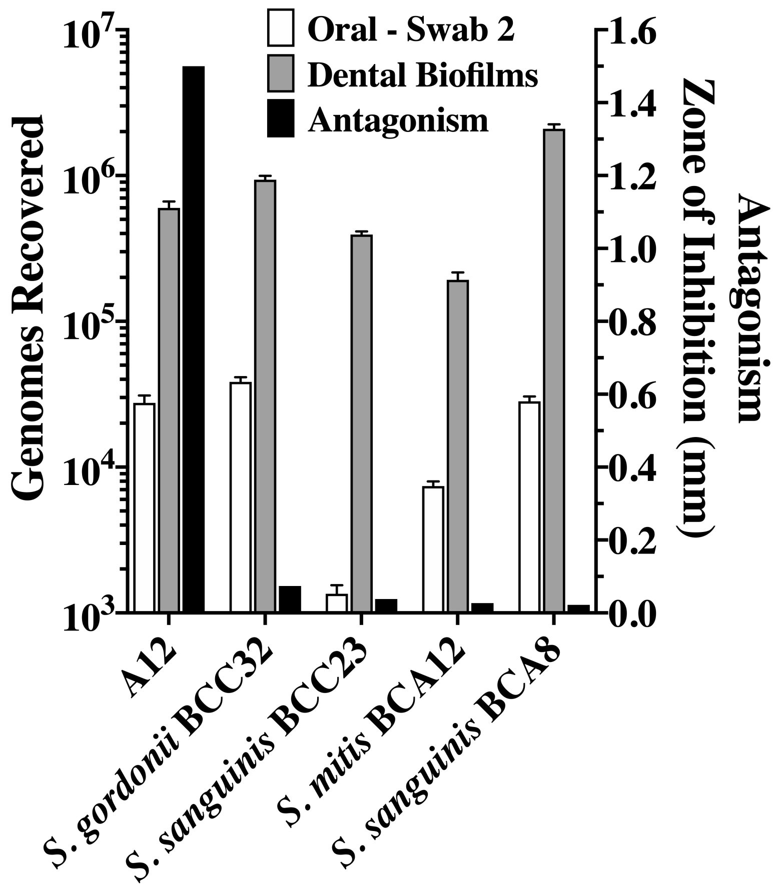

Fig. S3. Comparisons of antagonism and colonization between the five strains tested for competition against *S. mutans* with mice fed the high-sucrose diet plus arginine with 4% sucrose water. Values include colonization levels from swab 2 and dental biofilms observed by each strain alone as shown in Fig. 1 with mice fed the same diet. Also shown are values of antagonism against *Streptococcus mutans* UA159 characterized previously, *in vitro*, as described in Fig. S1.

Table S1. qPCR primers and run conditions.

S. intermedius A3	Reference Sequence: RJOL01000001.1
Target locus:	hypothetical protein, locus tag A3_00580
Forward primer:	5'-AACGGCTCAACAATCACAAA-3'
Reverse primer:	5'-GCTCATCTGCTCCATTTCCT-3'
Annealing/elongation:	69°C
Amplicon length:	145 bp
S. sp. A12	Reference Sequence: NZ_CP013651.1
Target locus:	hypothetical protein, locus tag ATM98_RS05545
Forward primer:	5'-CGCCTTCAAGACTATTGATATGATG-3'
Reverse primer:	5'-GGAAAACTAGCCCGTAAAGAATC-3'
Annealing/elongation:	68°C
Amplicon length:	124 bp
S. cristatus A52	Reference Sequence: RJPS01000003.1
Target locus:	Unannotated, putative ssp5. Between gene truB and locus tag A52_04160
Forward primer:	5'-GAGCGAATCATCAAGGATCAAAC-3'
Reverse primer:	5'-CGAGCAATAGCTTTCGTAATAGG-3'
Annealing/elongation:	68°C
Amplicon	131 bp
S. mitis BCC15	Reference Sequence: RJNH01000013.1
Target locus:	hypothetical protein, locus tag BCC15_00910
Forward primer:	5'-GGGCTTAGTAGACTTTCGTTA-3'
Reverse primer:	5'-GCCTCGATTTTATAGCTGT-3'
Annealing/elongation:	64°C
Amplicon length:	113 bp
S. sanguinis BCC23	Reference Sequence: RJMQ01000003.1
Target locus:	hypothetical protein, locus tag BCC23_05115
Forward primer:	5'-GCTGGATTCGGTTTGAGA-3'
Reverse primer:	5'-CCAAGACCCTAATCTCGTTT-3'
Annealing/elongation:	64.5 °C
Amplicon length:	151 bp
S. mitis BCC45	Reference Sequence: RJOB01000004.1
Target locus:	cpoA, locus tag BXX45_04665
Forward primer:	5'-GCTTACCGTGAATTAGTTCGTCTT-3'
Reverse primer:	5'-CCTGAGCGTTTCTTTTGGAATGT-3'
Annealing/elongation:	67°C
Amplicon length:	149 bp
S. mitis BCA12	Reference Sequence: RJNR01000002.1
Target locus:	cpoA, locus tag D8855_02495
Forward primer:	5'-GCTTACCGTGAATTAGTTCGTCTT-3'
Reverse primer:	5'-CCTGAGCGTTTCTTTTGGAATGT-3'
Annealing/elongation:	67°C
Amplicon length:	149 bp
S. sanguinis BCA8	Reference Sequence: JABBCN000000000.1
Target locus:	Unannotated region; *S. sanguinis* SK49 hypothetical protein, locus tag HMPREF9380_0379
Forward primer:	5'-CAGTTTGGTAGTTGTTCCTTC-3'
Reverse primer:	5'-TCTACTAACTCGCACTTCTAC-3'
Annealing/elongation:	66°C
Amplicon length:	125 bp
S. oralis sp. dentisani BCA1	Reference Sequence: RJVZ01000001.1
Target locus:	rebG, locus tag BCA1_00930
Forward primer:	5'-GACTGCTAAACGAGAGGATA-3'
Reverse primer:	5'-GCCGATGTATTTCGCTCTT-3'
Annealing/elongation:	64°C
Amplicon length:	146 bp
S. cristatus BCA6	Reference Sequence: RJPM01000001.1
Target locus:	ssp5_1, locus tag BCA6_01930
Forward primer:	5'-GAGCGAATCATCAAGGATCAAAC-3'
Reverse primer:	5'-CGAGCAATAGCTTTCGTAATAGG-3'
Annealing/elongation:	68°C
Amplicon length:	131 bp

Table S1. qPCR primers and run conditions, continued

S. mitis BCC08	Reference Sequence: RJPY01000002.1
Target locus:	cpoA, locus tag BCC08_03080
Forward primer:	5'-GCCTACCGTGAATTAGTTCGTCTT-3'
Reverse primer:	5'-CCTGAGCGTTTCTTTTGGAAGGT-3'
Annealing/elongation:	67°C
Amplicon length:	149 bp
S. gordonii BCC32	Reference Sequence: RJVX01000002.1
Target locus:	licB, locus tag BCC32_01205
Forward primer:	5'-CGCACGATAAATTGACAGA-3'
Reverse primer:	5'-CGCCATTCATCATACCATAA-3'
Annealing/elongation:	66°C
Amplicon length:	162 bp
S. mutans UA159	Reference Sequence: NC_004350.2
Target locus:	Locus tag SMU_292
Forward primer:	5'-TGGCAAGTCCTGATGGTTTGAC-3'
Reverse primer:	5'-GGAAGCGGAAGCTGTGATGAAC-3'
Annealing/elongation:	70°C
Amplicon length:	142 bp
Total bacteria	
Target locus:	*rpsL*, 30S ribosomal subunit protein S12
Forward primer:	5'-CCKAAYTCNGCNYTNCGTAA-3'
Reverse primer:	5'-CGHACMCCWGGWARGTCYTT-3'
Annealing/elongation:	55°C
Amplicon length:	149 - 153 bp

Reactions of 20 μl were run in a Bio-Rad CFX96 real-time PCR instrument using 10 μl SsoAdvanced Universal SYBR®Green Supermix (Bio-Rad, Hercules, CA), 0.5 μl each primer and 9 μl DNA. Samples were run at 3 min at 98°C followed by either 34 cycles (*rpsL*) or 40 cycles (98°C, 15 s; annealing/elongation temperature as indicated, 45 s; followed by a melt curve at 65-95°C at 0.5°C increments. All primers were used at 0.50 μM final concentration except for *S. sanguinis* BCC23 (0.15 μM) and rpsL (2.5 μM). Results were analyzed using the Bio-Rad CFX Manager program. Under the listed conditions, assays for human commensals failed to amplify *S. mutans* UA159 DNA or mouse oral commensal DNA. All work was performed in a BioSafety cabinet under aseptic conditions.

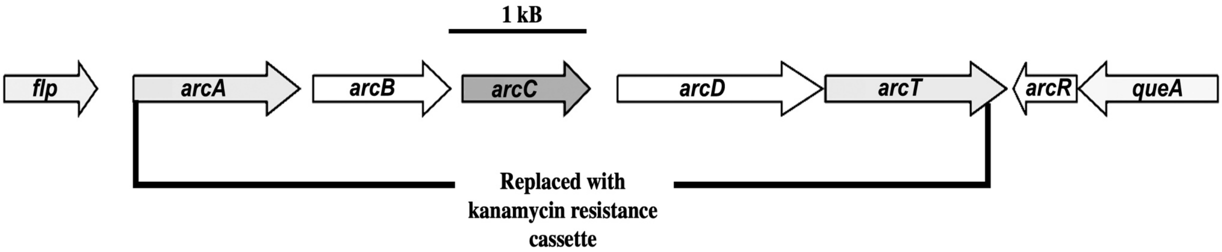

Fig. S4. Genomic region containing the ADS operon of A12, and indicating the region targeted for insertion of the kanamycin resistance cassette.

Table S2. Primers used in construction of A12 Δ*arcADS*

**Primer**	**Sequence (5' > 3')**
A12-*arc*ADS F-1	GGGGACTACTCCAGAAAGTACGG
A12-*arc*ADS R-2GA-Km	GCCATTTATTATTTCCTTCCTCTTTTACTATAGTCCTCCTTTTTT
A12-*arc*ADS F-3GA-Km	ATATTTTACTGGATGAATTGTTTTAGTAGACGACGATTAGCAGCC
A12-*arc*ADS R-4	GCCGACTCTGGTTGGACCAATATCTTTATC

Underlined sequences are those of the kanamycin resistance cassette.

Mice and caging

Three-week-old female specific pathogen-free BALB/cJ mice were ordered from The Jackson Laboratory (Ellsworth, ME) and paired in static microisolator cages within a cubicle of a BSL2 suite and with access to laboratory chow and sterile water ad libitum. The day after arrival, one mouse per pair underwent a single ear punch to the right ear to identify each mouse. A single 2.75 mm punch (BrainTree Scientific, Braintree, MA; Cat. #EP-SA 7075) was delivered in the middle of the right ear after first cleaning the site and the ear punch with an alcohol wipe. Cages contained a sterilized wired-bottom insert and sterile corn cob shavings underneath (approximately 6 mm depth). Wire cage bottoms reduce the tendency of mice to chew on underlying corn cob shavings that may become impacted within proximal dental spaces, thus interfering with bacteria-dental interactions, and further reduce coprophagic activity. A sterile nestlet made of pulped virgin cotton fiber (Lab Supply, Fort Worth, TX) was added to provide mice with a soft, inert and non-ingestible bedding material. Cages were changed twice a week and the water bottle changed weekly. Mice were weighed weekly throughout each experiment. To guard against cross-contamination between groups of mice and the introduction of human commensal bacteria from staff and investigators, all procedures with mice were carried out using ABSL-2 practices, and all materials in contact with mice were prepared under BSL2 conditions. Animals were euthanized in their home cage by CO_2 narcosis with subsequent cervical dislocation and decapitation. The University of Florida IACUC committee approved all animal procedures (IACUC Study #201509214).

Presentation of diets to mice

Diets were sterilized by gamma irradiation, received in powdered form in 2 kg packets and stored at 4°C. All diets were provided as a fine powder, as a small particle size was shown to prevent tooth fractures and to promote initiation of both smooth surface and sulcal carious lesions, presumably due to enhanced adhesiveness and to impaction, respectively (Konig 1962). Each diet was distributed to sterile 60 mm polystyrene petri dish bottoms or tops with approximately 13 g of powdered diet. Dishes were stacked in layers in sanitized buckets (5-quart plastic buckets with handle and lids (Cat. #80287 and #80288, United States Plastic Corp., Lima, OH) with sanitized heavy-duty plastic sheets between each layer, then stored at 4°C until distributed to cages. A sterile stainless steel disc (Fig. S5) was placed on top of the diet, then the dish inserted under a stainless steel dome (Unifab Corp., Portage, MI, Cat. #148-4-MFS-EP) with a 19 mm hole at the top to allow mice assess. We found empirically that addition of the disc and dome together lessened the tendency for mice to spread the diet throughout the cage and to contaminate the food with urine and feces, thus enhancing exposure of mice to uncontaminated diet. To prepare sucrose water, sucrose (≥ 99.5%; MilliporeSigma, St. Louis, MO) was dry-heat sterilized, dissolved in autoclaved Milli-Q water to 4% (w/v) and added to sterile water bottles within a BSL2 biosafety cabinet in the vivarium room.

Oral inoculations

Inoculants were prepared by culturing cells in 40 ml BHI broth, as described above, to an OD_{600} between 0.55 to 0.70. Cells were centrifuged (7,000 x *g*, 10 min at 4 °C), the cell pellet resuspended in 35 ml sterile ice-cold PBS followed by centrifugation (7,000 x *g*, 10 min at 4 °C). The cell pellet was resuspended in 24 ml sterile ice-cold PBS and cell concentration calculated from OD_{600} using the equation, cells/ml = (OD_{600} – 0.0831) / 6.57 x 10^{-10}, generated from a CFU growth curve of *S. mutans* UA159. A volume of resuspended cells equivalent to 2.3 x 10^{10} cells was centrifuged (7,000 x *g*, 10 min at 4 °C), the cell pellet resuspended in 4 ml sterile ice-cold PBS, then transferred to a 5 ml centrifuge tube and again centrifuged. The cell pellet was resuspended in 284 µl saliva buffer (50 mM KCl, 1.0 mM KPO_4, 0.35 mM K_2HPO_4, 1.0 mM $CaCl_2$ $2H_2O$, 0.1 mM $MgCl_2$ $6H_2O$, pH 6.5), transferred to a 2 ml tube followed by addition of 850 µl autoclaved 2.0% (w/v) carboxymethylcellulose (high viscosity, sodium salt; MilliporeSigma, St. Louis, MO; Cat #C5013) using a sterile 2.5 ml Eppendorf Combitip attached to a Eppendorf Repeater Pipette (Eppendorf North America, Inc., Hauppauge, NY). Repeated resuspensions with the positive displacement pipette served to resuspend the cells and then the tube capped. Once in the animal room the solution was drawn into a new sterile 2.5 ml Eppendorf Combitip and the Eppendorf Repeater Pipette adjusted to deliver 50 µl per injection (approximately 10^9 cells) for each mouse. Inoculations were carried out using a high viscosity solution of carboxymethylcellulose with the intent to increase the oral resident time of bacteria, and thus promote colonization.

Swabbing mice and recovery of bacteria

To swab mice, a sterile HydraFlock swab was inserted into the oral cavity between the lingual and palatal surfaces and rotated about 180 degrees in both directions. The swab was then repositioned between the buccal epithelium and teeth, then again rotated in both directions. This procedure was repeated on the opposite buccal side. The swab was immediately held over a 2 ml screw-cap sterile centrifuge tube preloaded with 1 ml sterile PBS (Thermo Fisher Scientific Inc., Waltham, MA; Cat #10010) and the handle cut aseptically about 25 mm above the tip, directing the tip into the PBS. The tube was capped and stored at -70 °C. To recover bacteria for DNA isolation, tubes were allowed to thaw on ice then vortexed 3 times for 5 seconds at the maximal setting of a Genie 2 (Model G-560) vortex with the standard cup top (Scientific Industries, Inc., Bohemia, NY). Swab tips were removed with stainless steel forceps after first sterilization using a Fisherbrand bacti-loop micro-sterilizer (Fisher Scientific Co., Pittsburgh, PA; Cat. #22-630-001), soaking 5s in 100% ice cold ethanol, shaking to remove excess ethanol and allowing to air dry while the next tube was prepared for tip extraction. The swab tip was lifted just above the PBS solution and lightly pressed against the side of the tube to allow entrapped solution to drain, then discarded. To enhance cell recoveries for DNA isolation, 200 µl of ice-cold PBS containing approximately 5 x 10^8 depurinated cells of laboratory strain *S. mitis* UF2 (see below) was immediately added. The tube was then vortexed 5 seconds, placed in a Nalgene benchtop cooler (Fisher Scientific Co.; Cat. #15-3550-62) pre-equilibrated to 4°C. Tubes were then centrifuged (10,000 x *g*, 10 min at 4 °C) to pellet recovered cells in the presence of depurinated carrier cells. Cell pellets were then processed for DNA isolation.

Recovery of bacteria from mandibular molars

To assess dental colonization, mandibles were aseptically extracted, tissue on the bone scraped away and the bone cut to about 3 mm proximal to the first and third molars. Molar teeth with attached bone were then sonicated in 1 ml of sterile ice-cold PBS in a siliconized 2 ml screw-capped sterile centrifuge tube placed in a well of an IsoFreeze Flipper 20-position tube rack (Scientific Specialties, Inc., Lodi, CA; Cat. #5610-40), pre-equilibrated to 4°C. The tube rack was placed in an ice bath mounted on a lab lift (Fisher Scientific, Waltham, MA; Cat. #02-216-477), then raised using the lift so that the probe of a Fisher F60 Sonic Dismembrator was inserted approximately 3/4th of the way into the PBS. The probe was first placed in an ice bath for 2 min then wiped down with 70% ethanol. The sample was sonicated using 6 pulses of 10 seconds at 20 Watts and 22.5 KHz with 90-second intervals between pulses, during which the tube rack was lowered to raise the probe above the sonicate. The tube rack was transferred to a biosafety cabinet and the molars with attached bone aseptically removed, then 200 µl of ice-cold PBS containing approximately 5 x 10^8 depurinated *S. mitis* UF2 cells immediately added. The tube was then vortexed and centrifuged as described above for cells recovered from swabs. Cell pellets were then processed for DNA isolation.

All tubes used for recovery of bacteria from swabs or molar plaque were sterile, made of polypropylene and siliconized to optimize cell recovery. Tubes were siliconized by adding 1 ml Sigmacote (MilliporeSigma; Cat. # SDSL2B), capping the tube, vortexing 2 s, removing Sigmacote, followed by washing with 1.5 ml of sterile-filtered and autoclaved Milli-Q water. Tubes were allowed to air dry overnight in a biosafety cabinet and then capped.

Quantitative PCR

Quantitative PCR was used to estimate recovered genomes of inoculated strains and of total recovered bacterial genomes in each DNA sample. To develop species-specific primers, genomic regions unique to a target streptococcal species were identified using VISTA (http://genome.lbl.gov/vista/index.shtml) and pre-computed full scaffold alignments at https://img.jgi.doe.gov. Putative unique regions of interest were then tested for alignments to all known complete and draft genomes of each species using BLASTN (https://blast.ncbi.nlm.nih.gov/Blast.cgi?PAGE_TYPE=BlastSearch&BLAST_SPEC=MicrobialGenomes) using the lowest stringency algorithm (e.g. "somewhat similar sequences"). Regions that aligned with high identity (≥95%) against numerous genomes of the target species, but not against any or few other streptococcal species, were tested further by BLASTN for alignments against complete and draft genomic sequences of representatives of other genera that in addition to streptococci are predominant in the oral microbiota of SPF mice (Rodrigue and Lavoie 1996, Chun, Kim et al. 2010, Culp, Robinson et al. 2015, Nicklas, Bisgaard et al. 2015, Abusleme, O'Gorman et al. 2020). Putative unique regions located within known or predicted coding regions of genes were evaluated for primers to generate amplicons of 150 bp or less

using MacVector (v 17.0). Primer pairs against 3-5 selected genes were then tested by qPCR using genomic DNA of the target species at increasing temperature and primer concentrations. Finally, primers were tested by qPCR for absence of amplification of genomic DNA from *S. mutans* UA159 and from oral swabs of untreated mice. Primers for *S. mutans* UA159 were likewise verified not to amplify each strain of human commensal streptococcus. All primers used are listed in Table S1.

To estimate total recovered bacteria, degenerate primers to conserved regions of the ubiquitous, single-copy gene, *rpsL* (30S ribosomal protein S12) (Lang, Darling et al. 2013). Note that targeting *16S rRNA* genes would only confound results due to variations in genome copy number among species. Shown in Table S3 are alignments of degenerate primers to the two conserved regions of *rpsL* genes as determined by ClustalW alignment (MacVector v.17.0) in multiple species of streptococci and representatives of other major genera combined account for at least 90% of the oral microbiota of SPF mice (Rodrigue and Lavoie 1996, Chun, Kim et al. 2010, Culp, Robinson et al. 2015, Nicklas, Bisgaard et al. 2015, Abusleme, O'Gorman et al. 2020). Also shown are alignments to the isolates of human streptococci tested in this study. Primers align very well in nearly all but a few cases, in which there is a single mismatched base, and with two base substitutions within the middle or near the 3'-end of the reverse primer for *Escherichia coli* and *Halomonas* species. Predicted amplicons are 149-153 bp, small enough for incorporation into a qPCR assay.

Standard curves were derived from DNA samples isolated from each strain grown to mid-exponential phase in BHI. *S. mutans* UA159 was used as standard for *rpsL* assays. DNA was quantified using the Qubit dsDNA HS assay (Thermo Fisher Scientific Inc, Waltham, MA) then diluted in 4 mM Tris-HCl, pH 8.0 to a concentration yielding 10^8 genomes in 125 µl, using each strain's genomic DNA for normalization. Subsequent dilutions of DNA representing 10^8 genomes were made to yield standards ranging typically from approximately 500 to10^8 genomes in 125 µl (equivalent to the original volume of isolated DNA) for assays of human commensal streptococci or 10^3 to 10^7 genomes in 125 µl for *rpsL* assays. All dilutions were performed in siliconized 2 ml tubes. Efficiencies, slopes and R^2 values for standard curves for streptococci were greater than 90%, -3.244 and 0.972, respectively. A standard curve for *S. cristatus* A52 is shown in Fig. S6.

Recovery of murine autochthonous oral bacteria was estimated by subtracting the number of total recovered genomes of inoculated strains from the total number of recovered bacterial genomes from the *rpsL* assay.

Preparing jaws for caries scoring

Flesh from frozen skulls with maxillary jaws were removed using a colony of Dermestid beetles. Once thawed, the outer skin was pulled away and the skulls placed in a cubicle of a homemade Lucite tray with small holes at the bottom of each cubical wall to allow beetles to gain access (see Fig. S7A and S5B). After 2-3 days skulls were removed and placed in repeated changes of 2% ammonium hydroxide for 30 min each until the solution was cleared, then stored in 2% formalin. Skulls and mandibular molars were rinsed in water, air dried and stained with Murexide (Baker #R372-01 Murexide monohydrate powder; 0.024% w/v in 70% ethanol) in an airtight container for 16-18 h at room temperature for visualization of exposed dentin during subsequent caries scoring. Skulls and mandibular molars were collected in a small kitchen strainer, then washed under a heavy stream of water and allowed to air dry for at least 48 h. Maxillary and mandibular molars were then scored for smooth surface caries and their severities on buccal, lingual and proximal surfaces with the aid of an Olympus dissecting microscope (Model SZX16). To score sulcal caries and their severities, molars were hemisectioned along the midline of their apical surface in a mesiodistal sagittal plane to allow viewing a cross-section of the enamel, dentin and pulpal layers. Skulls and mandibular molars were first embedded in epoxy (Embed-It Low Viscosity Epoxy Kit; Polysciences, Inc., Warrington, PA; Cat. #24300) to a level about 1 mm below the enamel crown using silicone molds. For mandibular molars, an embedding mold with 14 X 5 X 4 mm deep cavities (Electron Microscopy Sciences, Hatfield, PA; Cat. #70900) was used, and for skulls a mold with 28.6 X 17.5 X 12.7 mm deep cavities (Pastry Chef Central, Inc., Boca Raton, FL; Cat # SM-SF181). Mandibular molars were first stabilized in position with a drop of superglue, then 140 µl of epoxy solution added. For skulls, 1.4 ml epoxy was added. Molds were then incubated for 16 to 28 h at 64.5 °C for complete polymerization and curing, followed by > 24 h at room temperature to finish hardening. Epoxy blocks were mounted in a custom-made vice attached to a micromanipulator and molars sectioned with an abrasive disk attached to a hand piece mounted on a ball-bearing slider (see Fig S7C-F).

References

Abusleme, L., H. O'Gorman, N. Dutzan, T. Greenwell-Wild and N. M. Moutsopoulos (2020). "Establishment and Stability of the Murine Oral Microbiome." J Dent Res **99**(6): 721-729.

Chun, J., K. Y. Kim, J. H. Lee and Y. Choi (2010). "The analysis of oral microbial communities of wild-type and toll-like receptor 2-deficient mice using a 454 GS FLX Titanium pyrosequencer." BMC Microbiol **10**: 101.

Culp, D. J., B. Robinson, M. N. Cash, I. Bhattacharyya, C. Stewart and G. Cuadra-Saenz (2015). "Salivary mucin 19 glycoproteins: innate immune functions in Streptococcus mutans-induced caries in mice and evidence for expression in human saliva." J Biol Chem **290**(5): 2993-3008.

Konig, K. G. (1962). "Effects of particle size of corn and sugar diets and of mastication on caries incidence in Osborne-Mendel rats." J Dent Res **41**: 966-985.

Lang, J. M., A. E. Darling and J. A. Eisen (2013). "Phylogeny of bacterial and archaeal genomes using conserved genes: supertrees and supermatrices." PLoS One **8**(4): e62510.

Nicklas, W., M. Bisgaard, B. Aalbaek, P. Kuhnert and H. Christensen (2015). "Reclassification of Actinobacillus muris as Muribacter muris gen. nov., comb. nov." Int J Syst Evol Microbiol **65**(10): 3344-3351.

Rodrigue, L. and M. C. Lavoie (1996). "Comparison of the proportions of oral bacterial species in BALB/c mice from different suppliers." Lab Anim **30**(2): 108-113.

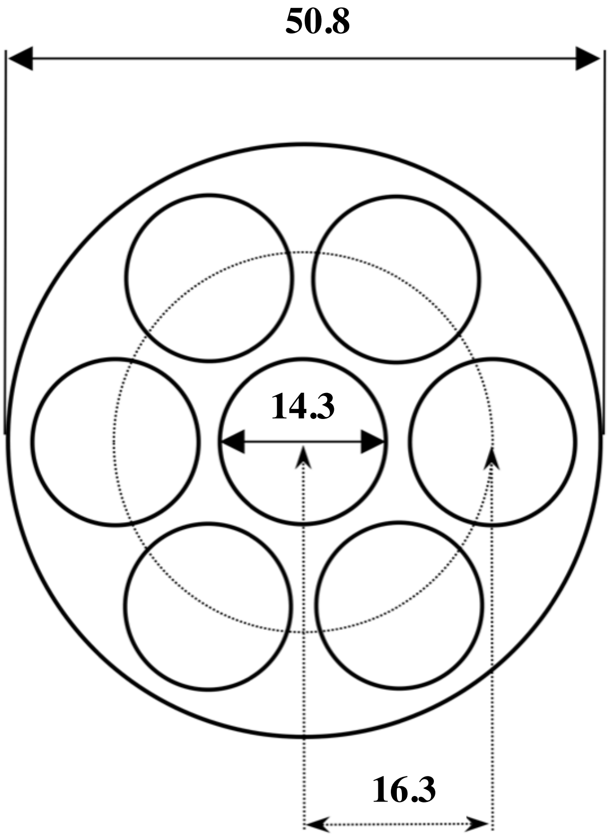

Fig. S5. Diagram of 12 gauge stainless steel (Type 316L) disc used to partially cover powdered diets within 60 mm Petri dishes. The outer holes are arranged at 60° angles from the center of the disc. All measurements are in mm.

Table S3. Comparisons of binding sites for rpsL primers in representatives of oral bacterial genera and species identified in SPF laboratory mice and in human oral streptococci used in this study. Shown are the positive strands of *rpsL* genes that align to forward and reverse degenerate primers. Nucleotides in bold with yellow highlight are not complementary to the indicated primer.

***rpsL* Primers: Forward and Reverse Complement of Reverse**	**CCKAAYTCNGCNYTNCGTAA**	**AARGACHTWCCWGGKGTDCG**
**Species (Accession Number)**		
Actinobacillus succinogenes 130Z (NC_009655.1)	CCGAACTCAGCATTACGTAA	AAAGACTTACC**G**GGTGTGCG
Corynebacterium renale CIP 52.96 (NZ_LDYE00000000.1)	CCTAACTCTGCACTGCGTAA	AAGGACCT**C**CCAGGTGTTCG
Corynebacterium stationis GA-15 (NZ_LSTQ00000000.1)	CCTAACTCTGCTCTCCGTAA	AAGGACCTCCCAGGTGTTCG
Corynebacterium mastitidis DSM 44356 (NZ_AQXB00000000.1)	CCTAACTCCGCTCTGCGTAA	AAAGACCTTCCTGGTGTTCG
Enterobacter cloacae PIMB10EC27 (NZ_CP020089.1)	CCAAACTCCGCACTGCGTAA	AAAGACCTTCC**G**GGTGTTCG
Enterococcus faecalis V583 (NC_004668)	CCGAACTCAGCTTTACGTAA	AAAGACTTACCAGGGGTACG
Escherichia coli str. K-12 substr. MG1655 (NC_000913.3)	CCGAACTCCGCGCTGCGTAA	AAAGACCT**C**CC**G**GGTGTTCG
Halomonas alkaliphila X3 (NZ_CP024811.1)	CCGAACTCGGCCCTTCGTAA	AAGGA**T**TT**G**CCAGGTGTGCG
Halomonas venusta MA-ZP17-13 (NZ_CP034367.1)	CCGAACTCGGCCCTGCGTAA	AAGGA**T**TT**G**CCAGGTGTGCG
Lactobacillus murinus ASF361 (AQFS00000000.1)	CCTAACTCTGCTTTACGTAA	AAAGACTTACCAGGGGTTCG
Lactobacillus murinus salivarious ZLS006 (NZ_CP020858.1)	CCTAACTCTGCTTTACGTAA	AAAGACTTACCTGGGGTTCG
Muribacter muris Ackerman80-443D (JWIZ00000000.1)	CCTAACTCAGCGTTACGTAA	AAAGACTTACC**G**GGTGTGCG
Streptococcus sp. 2_1_36FAA (NZ_GG704939)	CCTAACTCTGCCCTTCGTAA	AAAGACCTTCCAGGGGTACG
Streptococcus A12 (NZ_CP013651.1)	CCGAACTCTGCCCTTCGTAA	AAAGACCTTCCAGGGGTACG
Streptococcus sp. M143 (NZ_ACRK00000000.1)	CCTAACTCTGCCCTTCGTAA	AAAGACCTTCCAGGGGTACG
Streptococcus acidominimus NCTC11291 (NZ_LT906454.1)	CCTAACTCTGCCCTTCGTAA	AAGGACCTTCCAGGGGTACG
Streptococcus cristatus ATCC 51100 (NZ_LS483383.1)	CCTAACTCAGCCCTTCGTAA	AAAGACCTTCCAGGGGTACG
Streptococcus danieliae NM51_B2-22 (WSRS01000036.1)	CCAAACTCAGCCCTTCGTAA	AAGGACCTTCCAGGGGTACG
Streptococcus oralis subsp. dentisani F0392 (NZ_CP034442.1)	CCTAACTCAGCCCTTCGTAA	AAAGACCTTCCAGGGGTACG
Streptococcus equi equi 404 (NC_012471)	CCTAACTCAGCCCTTCGTAA	AAGGACCTTCCAGGGGTACG
Streptococcus galloyticus UCN34 (NC_013798.1)	CCTAACTCAGCCCTTCGTAA	AAAGACCTTCCAGGGGTACG
Streptococcus gordonii Challis CH1 (NC_009785)	CCTAACTCTGCCCTTCGTAA	AAAAGACCTTCCAGGGGTAC
Streptococcus intermedius ATCC 15912 (BASV00000000.1)	CCGAATTCGGCTCTTCG**C**AA	AAAGACCTTCCAGGGGTACG
Streptococcus intermedius B196 (NC_022246.1)	CCGAATTCGGCTCTTCGTAA	AAAGACCTTCCAGGGGTACG
Streptococcus macacae NTC 11558 (NC_022246.1)	CCTAACTCAGCGCTTCGTAA	AAAGACCTTCCAGGGGTACG
Streptococcus marmotae HTS5 (NZ_CP015196)	CCTAACTCAGCCCTTCGTAA	AAAGACCTTCCAGGGGTACG
Streptococcus mitis B6 (NC_013853)	CCTAACTCAGCCCTTCGTAA	AAAGACATTCCAGGGGTACG
Streptococcus mutans NN2025 (NC_013928)	CCTAACTCTGCTCTTCGTAA	AAGGACCTTCCAGGGGTACG
Streptococcus mutans UA159 (NC_004350.2)	CCTAACTCTGCTCTTCGTAA	AAGGACCTTCCAGGGGTACG
Streptococcus oralis UO5 (NC_015291)	CCTAACTCTGCCCTTCGTAA	AAAGACCTTCCAGGGGTACG
Streptococcus parasanguinis ATCC 15912 (NC_015678.1)	CCTAACTCTGCCCTTCGTAA	AAAGACCTTCCAGGGGTACG
Streptococcus respiraculi HTS25 (NZ_CP022680)	CCTAACTCAGCCCTTCGTAA	AAAGACCTTCCAGGGGTACG
Streptococcus salivarius NCTC 8618 (NZ_CP009913.1)	CCTAACTCAGCCCTTCGTAA	AAAGACCTTCCAGGGGTACG
Streptococcus sanguinis NCTC11086 (NZ_LS483364)	CCAAACTCTGCCCTTCGTAA	AAAGACCTTCCAGGGGTACG
Streptococcus sobrinus ATCC 33478 (AOCE01000060)	CCTAACTCTGCCCTTCGTAA	AAAGACCTTCCAGGGGTTCG
Streptococcus thoraltensis DSM 12221 (ARCI01000027.1)	CCTAACTCAGCCCTTCGTAA	AAAGACCTTCCAGGGGTACG
Staphylococcus saprophyticus 772 (NZ_JUTO00000000.1)	CCTAACTCAGCTTTACGTAA	AAAGACTTACCTGGTGTGCG
Staphylococcus xylosus HKUOPL8 (NZ_CP007208.1)	CCTAACTCCGCGTTACGTAA	AAAGACTTACCTGGTGTGCG
Staphylococcus cohnii subsp. urealyticus DSM 6718 (PPRL01000025.1)	CCTAACTCTGCGTTACGTAA	AAAGACTTACCTGGTGTGCG
**Human Commensal Streptococci in this Study**		
Streptococcus intermedius A3 (RJOL01000000)	CCGAATTCGGCTCTTCGTAA	AAAGACCTTCCAGGGGTACG
Streptococcus A12 (NZ_CP013651.1)	CCGAATTCGGCTCTTCGTAA	AAAGACCTTCCAGGGGTACG
Streptococcus cristatus A52 (RJPS01000000)	CCTAACTCAGCCCTTCGTAA	AAAGACCTTCCAGGGGTACG
Streptococcus oralis subsp. dentisani BCA1 (RJVZ01000000)	CCTAACTCTGCCCTTCGTAA	AAAGACCTTCCAGGGGTACG
Streptococcus cristatus BCA6 (RJPM01000000)	CCTAACTCAGCCCTTCGTAA	AAAGACCTTCCAGGGGTACG
Streptococcus sanguinis BCA8 (JABBCN000000000)	CCGAACTCTGCCCTTCGTAA	AAAGACCTTCCAGGGGTACG
Streptococcus mitis BCA12 (RJNR01000000)	CCTAACTCTGCCCTTCGTAA	AAAGACCTTCCAGGGGTACG
Streptococcus mitis BCC08 (RJPY01000000)	CCTAACTCAGCCCTTCGTAA	AAAGACCTTCCAGGGGTACG
Streptococcus mitis BCC15 (RJNH01000000)	CCTAACTCAGCCCTTCGTAA	AAAGACCTTCCAGGGGTACG
Streptococcus sanguinis BCC23 (RJMQ01000000)	CCGAACTCTGCCCTTCGTAA	AAAGACCTTCCAGGGGTACG
Streptococcus gordonii BCC32 (RJVX01000000)	CCTAACTCTGCCCTTCGTAA	AAAGACCTTCCAGGGGTACG
Streptococcus mitis BCC45 (RJOB01000000)	CCTAACTCAGCCCTTCGTAA	AAAGACCTTCCAGGGGTACG

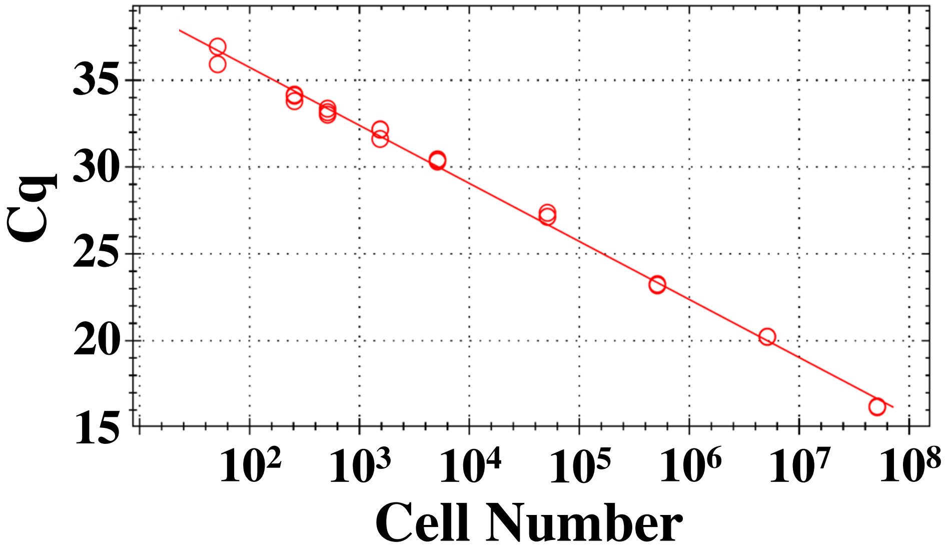

Fig. S6. Standard curve of qPCR assay for *S. cristatus* A52. Efficiency = 89.3%, slope = -3.714, y-intercept = 44.99 and $R^2 = 0.997$.

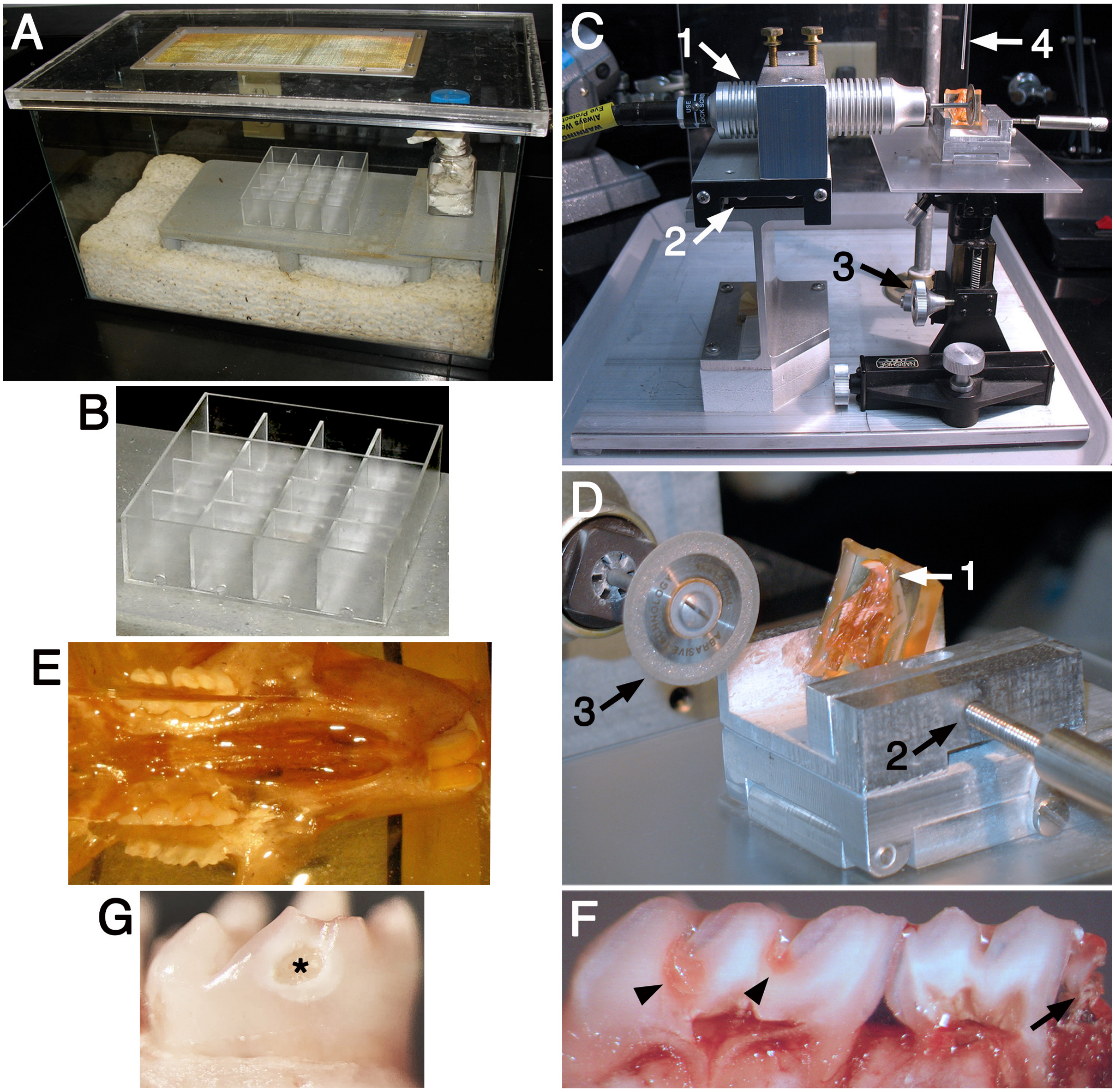

Fig. S7. Preparations for caries scoring. **A.** Dermestid beetle colony for defleshing skulls and mandibles. A fiberglass platform stands over the cotton nesting area and supports a Lucite tray with separate cubicles for each skull. **B.** Closeup of the Lucite tray. Each cubicle wall a has small hole at the bottom for beetles to gain access. A bottle with water and a paper wick provides moisture for the beetles. **C.** Apparatus used to slice molars for sulcal scoring. A drill motor (1; Foredom M.SRM bench motor with H.44T hand piece; Foredom Electric Co., Bethel, CT) with the handpiece mounted on a ball-bearing slider (2) for controlled, but restricted front-to-back movement of the motor and cutting disc. The specimen is mounted into a vice connected to a platform on a micromanipulator (3; Narishige International USA, Inc., Amityville, NY). During cutting at 18,000 rpm, water rapidly drips from a stainless steel tube (4) for cooling, powered by a small peristaltic pump. Not shown is a Lucite shield placed in front of the operator when cutting to collect water spray. **D.** A close-up view of a maxilla partially embedded in epoxy (1) and mounted into the vice (2). The double-sided diamond coated disk (3; Thin-Flex X927-7 0.15 mm thick abrasive disc, Abrasive Technology, Lewis Center, OH) is guided into the aligned teeth by gliding the drill motor by hand. Dental loupes are used to lineup the teeth to the disc for a midline cut. **E.** The left and right maxillary molars are shown after sectioning through the midline and further separating the two halves by prying using the side walls of a razor blade. **F.** View of sectioned maxillary molars stained with murexide. The third molar is mostly degraded (arrow). Lesions deep into the dentine in the first molar are stained red (arrowhead), especially in the anterior fissure. **G.** Smooth surface caries on the buccal surface of a first molar. Asterisk indicates lesion into the dentine, surrounded by dull white enamel caries.
